# W361R mutation in GaaR, the regulator of D‐galacturonic acid‐responsive genes, leads to constitutive production of pectinases in *Aspergillus niger*

**DOI:** 10.1002/mbo3.732

**Published:** 2018-10-08

**Authors:** Ebru Alazi, Jing Niu, Simon B. Otto, Mark Arentshorst, Thi T. M. Pham, Adrian Tsang, Arthur F. J. Ram

**Affiliations:** ^1^ Molecular Microbiology and Biotechnology Institute of Biology Leiden Leiden University Leiden The Netherlands; ^2^ Centre for Structural and Functional Genomics Concordia University Montreal Québec, Canada

**Keywords:** *Aspergillus niger*, constitutively active transcription factor, CRISPR‐Cas9, missense mutation, pectinase, transcription factor localization

## Abstract

Polysaccharides present in plant biomass, such as pectin, are the main carbon source for filamentous fungi. *Aspergillus niger* naturally secretes pectinases to degrade pectin and utilize the released monomers, mainly D‐galacturonic acid. The transcriptional activator GaaR, the repressor of D‐galacturonic acid utilization GaaX, and the physiological inducer 2‐keto‐3‐deoxy‐L‐galactonate play important roles in the transcriptional regulation of D‐galacturonic acid‐responsive genes, which include the genes encoding pectinases. In this study, we described the mutations found in *gaaX* and *gaaR* that enabled constitutive (i.e., inducer‐independent) expression of pectinases by *A. niger*. Using promoter‐reporter strains (P*pgaX*‐*amdS*) and polygalacturonic acid plate assays, we showed that W361R mutation in GaaR results in constitutive production of pectinases. Analysis of subcellular localization of C‐terminally eGFP‐tagged GaaR/GaaR^W^ ^361R^ revealed important differences in nuclear accumulation of N‐ versus C‐terminally eGFP‐tagged GaaR.

## 1 INTRODUCTION

Polysaccharides present in plant biomass, such as pectin, are the principle carbon source for saprophytic filamentous fungi like *Aspergillus niger* and *Neurospora crassa* (Kowalczyk, Benoit, & de Vries, 2014; Znameroski & Glass, 2013). In plant pathogenic fungi, such as *Botrytis cinerea*, pectin degradation is considered an important virulence event as the fungus has to degrade the pectin that is part of the cell wall in order to invade the plant cell (Choquer et al., 2007; Zhang & van Kan, 2013). Different plants produce different kinds of pectins which have been categorized in four main substructures with increasing complexity. All four pectin substructures (i.e., polygalacturonic acid (PGA), xylogalacturonan, rhamnogalacturonan I, and rhamnogalacturonan II) contain D‐galacturonic acid (GA) in their backbones, with various monomers or polymers attached to their backbones. PGA is the major polysaccharide present in pectin and consists of α‐1,4‐linked GA subunits (Caffall & Mohnen, 2009). When *A. niger* encounters pectin in its environment, it secretes pectinases to degrade pectin, transports the released monosaccharides into the cell and catabolizes them to generate energy. The expression of GA‐induced genes encoding pectinases and enzymes involved in the catabolism of GA are tightly regulated. In *A. niger*, a GA‐responsive element (GARE) was identified in the promoter region of GA‐induced genes (Martens‐Uzunova & Schaap, 2008) and mutational analysis of this element (5′‐CCNCCAA‐3′) showed its requirement for induction in response to the presence of GA (Niu et al., 2015). The same GARE element was also identified in the promoter regions of GA‐induced genes in *B. cinerea* (Zhang et al., 2016), indicating a conserved transcriptional control mechanism. A yeast one‐hybrid screen was conducted in *B. cinerea* which resulted in the identification of a fungal GA‐responsive Zn_2_Cys_6_ type transcription factor named GaaR (Zhang et al., 2016). The *A. niger* GaaR orthologue was identified via its homology to the *B. cinerea* GaaR and was shown to be required for growth on GA and polygalacturonic acid (Alazi et al., 2016). GaaR was found to be essential for the GA‐responsive expression of several pectinases, (putative) GA‐transporters, and GA catabolic pathway enzymes (Alazi et al., 2016). The gene encoding GaaR is present in the genomes of Ascomycetes belonging to the Pezizomycotina subdivision which includes members of Eurotiomycetes (*Aspergillus*,* Penicillium*, and *Talaromyces* spp.), Leotiomycetes (*Botrytis* and *Oidiodendron* spp.), Sordariomycetes (*Neurospora*,* Myceliophthora*,* Trichoderma*, and *Fusarium* spp.), and Dothideomycetes (*Zymoseptoria* and *Aureobasidium* spp.). The function of GaaR has not been studied yet in species apart from *A. niger* and *B. cinerea*. In *N. crassa,* the PDR‐1 transcription factor is important for growth on PGA and pectin (Thieme et al., 2017). Phylogenetically, PDR‐1 is the ortholog of the *A. niger* rhamnose regulator RhaR. However, the Δ*rhaR* mutant of *A. niger* has no apparent growth defect on PGA or pectin (Ram et al., unpublished results; Gruben et al., 2014), indicating that expression of pectinases in *A. niger* and *N. crassa* is at least partially under control of different regulators.

*Aspergillus niger* mutants showing constitutive production of pectinases were previously obtained via a forward genetic screen using the promoter–reporter strain P*pgaX*‐*amdS ∆creA* (JN29.2) (Niu et al., 2017). P*pgaX*‐*amdS ∆creA* (JN29.2) is able to grow on acetamide as the sole nitrogen source only when the GA‐responsive promoter of the *pgaX* (NRRL3_03144) gene is activated to express the *amdS* gene that encodes the acetamide catabolic enzyme acetamidase (Niu et al., 2015). In total, 64 mutants showing constitutive production of pectinases were isolated on plates containing minimal medium (MM) with glucose as the carbon source and acetamide as the sole nitrogen source. The genomes of five mutants were sequenced and it was found that they carry allelic mutations in a common gene, which was named the repressor of D‐galacturonic acid utilization, *gaaX* (NRRL3_08194) (Niu et al., 2017). Transcriptome analysis revealed that deletion of *gaaX* results in the constitutive expression of pectinase genes (Niu et al., 2017). GaaX is suggested to inhibit GaaR function by stoichiometric binding to GaaR under non‐inducing conditions (Alazi et al., 2018; Niu et al., 2017). Interestingly, the genes encoding the repressor protein (GaaX) and the transcriptional activator (GaaR) are located side‐by‐side on the chromosome of *A. nige*r as well as in other Pezizomycotina species (Niu et al., 2017). The clustering of genes encoding the GA‐specific transcriptional activator (GaaR) and the repressor protein (GaaX) is similar to that of the activator‐repressor modules *qutA*‐*qutR* and *qa‐1F*‐*qa‐1S* involved in the utilization of the plant cell wall constituent quinic acid in *Aspergillus nidulans* and *N. crassa*, respectively. In both the quinic acid and the GA utilization systems, deletion of the repressor results in constitutive expression of target genes which requires the presence of the corresponding Zn_2_Cys_6_ type transcriptional activator (Giles et al., 1985; Grant, Roberts, Lamb, Stout, & Hawkins, 1988; Lamb et al., 1996; Niu et al., 2017). Moreover, *A. nidulans* strains expressing multiple copies of *qutA* showed constitutive expression of the genes involved in quinic acid utilization (Lamb et al., 1996). Similarly, overexpression of *gaaR* in *A. niger* resulted in constitutive expression of the genes involved in the breakdown of pectin and utilization of GA (Alazi et al., 2018). These results indicate that the activity of GaaR is inhibited by GaaX under non‐inducing conditions possibly via protein–protein interaction and that GA‐responsive gene expression only requires the presence of active GaaR relieved/escaped from GaaX inhibition. This suggests that the inducer binds to GaaX resulting in a GaaX‐unbound and active form of GaaR under inducing conditions. The catabolic intermediate 2‐keto‐3‐deoxy‐L‐galactonate produced from GA was shown to induce the production of pectinases by *A. niger* which makes it reasonable to suggest that 2‐keto‐3‐deoxy‐L‐galactonate binds to GaaX resulting in the dissociation of GaaX from GaaR, thereby releasing GaaR as an active transcription factor (Alazi et al., 2017). Because overexpression of the activators involved in GA or quinic acid utilization has similar effects, it is likely that activation mechanism of both QutA/QutR and GaaR/GaaX is conserved and comparable.

Pectinases produced by *A. niger* are used industrially (Edwards & Doran‐Peterson, 2012; Kashyap, Vohra, Chopra, & Tewari, 2001; Khan, Nakkeeran, & Umesh‐Kumar, 2013; Toushik, Lee, Lee, & Kim, 2017), especially in food industry and in hydrolysis of plant biomass for the subsequent production of biofuel and high‐value biopolymers. Inducer‐independent production of pectinases by *A. niger* would enable the use of waste and residues from agriculture, forestry, and food industries as a cheap and sustainable feedstock for fungal cultivations. In this study, we describe mutations in *gaaX* and *gaaR* that result in constitutive production of pectinases by *A. niger*. We show that a gain‐of‐function mutation in *gaaR* causing an amino acid change from tryptophan to arginine at position 361 (GaaR^W361R^) leads to a constitutively active form of the GaaR transcription factor, leading to inducer‐independent production of pectinases.

## 2 MATERIALS AND METHODS

### 2.1 Strains, media, and growth conditions

All strains used in this study are listed in Table S1. Media were prepared as described in Arentshorst, Ram, and Meyer (2012). Radial growth phenotype of the strains was analyzed on minimal medium (pH 5.8) containing 1.5% (w/v) agar (Scharlau, Barcelona, Spain) and various carbon sources: 50 mM D‐glucose (VWR International, Amsterdam, The Netherlands), D‐fructose (Sigma‐Aldrich, Zwijndrecht, The Netherlands) or GA (Chemodex, St Gallen, Switzerland); or 1% (w/v) PGA (Sigma‐Aldrich) or apple pectin (AP) (Sigma‐Aldrich). Media of the uridine auxotrophic strains were supplemented with 10 mM uridine. Plates were inoculated with 5 μl 0.9% NaCl containing 1 × 10^4^ or 5 × 10^4^ freshly harvested spores, and cultivated at 30°C for 7 or 5 days, respectively. MM (pH 5.8) containing 1.5% (w/v) agar, 10 mM acetamide (Sigma‐Aldrich, Steinheim, Germany) as the sole nitrogen source, and glucose, D‐fructose, GA or 2‐keto‐3‐deoxy‐L‐galactonate as the carbon source was prepared as described previously (Arentshorst, Ram, and Meyer, 2012). Plates were inoculated with 5 μl 0.9% NaCl containing 10^4^ freshly harvested spores and cultivated at 30°C for 7 or 10 days. Filter‐sterilized carbon source solutions were added after autoclaving MM containing agar. PGA and AP were autoclaved together with the medium. All growth experiments were performed in duplicate.

For enzymatic analysis, 300 ml shake flasks that include 100 ml MM (pH 5.8) containing 50 mM D‐fructose and 0.003% yeast extract were inoculated with 7.5 × 10^7^ freshly harvested spores and cultivated for 36 hr in a rotary shaker at 30°C and 250 rpm. Experiments were performed in duplicate.

For microscopic analysis of the localization of the eGFP‐tagged GaaR or GaaR^W361R^ proteins, 4 × 10^5^ freshly harvested spores were inoculated on cover slips in Petri dishes that include 4 ml MM containing 0.003% yeast extract and 10 mM D‐fructose or 2‐keto‐3‐deoxy‐L‐galactonate, and grown at 30°C for approximately 21 hr. For each condition, two biological replicates were performed.

### 2.2 Sequencing *gaaX* and *gaaR* genes from mutants displaying constitutive production of enzymes involved in PGA utilization

Sixty‐four *trans*‐acting mutants spontaneous or obtained after mild UV mutagenesis with constitutive production of enzymes involved in PGA utilization were obtained as previously described (Niu et al., 2017) on solid MM containing 50 mM glucose as the carbon source and 10 mM acetamide as the sole nitrogen source. Genomic DNA of 11 spontaneous and 53 UV mutants was extracted as described by Arentshorst, Ram, and Meyer (2012), and *gaaX* and/or *gaaR* genes were PCR‐amplified using the primers gaaX_P5f and gaaX_GSP9r, or gaaRP7f and gaaRP8r, respectively (Table S2). The PCR fragments were sequenced in both directions using *gaaX* or *gaaR* sequencing primers, respectively (Table S2).

### 2.3 Construction of the promoter–reporter strains expressing *gaaR* ^*W361R*^

Protoplast‐mediated transformation of *A. niger* and purification of the transformants were performed as described by Arentshorst, Ram, and Meyer (2012).

The *gaaR* ^*W361R*^ gene together with its 964‐bp promoter and 992‐bp terminator regions was amplified by PCR using the primers gaaRP5f and gaaRP6r (Table S2) with JN103.1 genomic DNA as template. The PCR product was transformed into strains JC1.5 and JN29.2, yielding in the strains JN130.4 and JN129.1, respectively. Transformants were selected on plates (Arentshorst et al., 2012) containing 10 mM acetamide as the sole nitrogen source. Correct gene replacements in strains JN130.4 and JN129.1 were verified by Southern blot and sequencing analyses. For sequence analysis, the *gaaR* locus was amplified using the primers gaaRP7f and gaaRP8r (Table S2) and JN130.4 and JN129.1 genomic DNA as template, and ligated into pJET1.2/blunt cloning vector (Thermo Fisher Scientific, Carlsbad, CA). The resulting plasmids were amplified in *Escherichia coli* and sequenced using *gaaR* sequencing primers. Integration of the *gaaR* ^*W361R*^ into the endogenous *gaaR* locus was confirmed via Southern blot analysis. Genomic DNA was digested overnight with *Nco*I or *Hin*dIII restriction enzymes. A 501‐bp fragment containing the *gaaR* gene was PCR‐amplified using the primer pairs listed in Table S2 with N402 genomic DNA as template and was used as a probe.

### 2.4 Construction of strains expressing *gaaR‐eGFP* or *gaaR‐(GA)*_*4*_ *‐eGFP*

The plasmid pEA9 containing the P*gaaR*‐*gaaR*‐*eGFP*‐T*gaaR* construct was created as follows: The *eGFP* gene and the 715‐bp terminator of the *gaaR* gene were amplified by PCR using the primer pairs listed in Table S2 with the plasmid pFG029 (unpublished vector, containing P*gpdA*‐*eGFP*‐T*trpC*) and N402 genomic DNA as template, respectively. *eGFP* and T*gaaR* were combined by fusion PCR using primers GaaR_GFP1F and GaaR_GFP3R. The *gaaR* gene without the stop codon and together with the 731‐bp promoter region was PCR amplified using the primer pairs listed in Table S2. P*gaaR*‐*gaaR* and *eGFP*‐T*gaaR* were combined by fusion PCR using primers eGFP‐gaaR‐For and eGFP‐gaaR‐Rev, ligated into pJET1.2/blunt cloning vector (Thermo Fisher Scientific) and amplified in *E. coli*. Sequencing of pEA9 showed that a PCR error (T to C) has occurred in T*gaaR* at a distance of 519 bp downstream of the stop codon of the *eGFP* gene.

The plasmid pEA10 containing the P*gaaR*‐*gaaR‐(GA)*_*4*_‐*eGFP*‐T*gaaR* construct was created in a similar way to pEA9, except that P*gaaR*‐*gaaR‐(GA)*_*4*_ was amplified using the primer GaaR_GFP6R adding a linker containing four repeats of glycine and alanine residues between GaaR and eGFP. Sequencing of pEA10 showed that a PCR error (C to T) has occurred in T*gaaR* at a distance of 430 bp downstream of the stop codon of the *eGFP* gene.

To create the strain EA29.14, pEA9 was co‐transformed into strain JN36.1 (*ΔgaaR*) together with the plasmid pMA357 containing the *A. nidulans amdS* gene behind the *A. nidulans gpdA* promoter (Alazi et al., 2016). EA30.6 was created by co‐transformation of pEA10 into strain JN36.1 (Δ*gaaR*) together with the plasmid pMA357. Transformants were selected on transformation plates containing acetamide as the sole nitrogen source.

### 2.5 Construction of the Δ*gaaR*::*AOpyrG* deletion strain

SO1.1 (Δ*gaaR::AOpyrG*) was created using the split marker approach (Arentshorst, Niu, & Ram, 2015). 5′ and 3′ flanks of *gaaR* gene were PCR‐amplified using the primer pairs listed in Table S2 with N402 genomic DNA as template. The *Aspergillus oryzae pyrG* gene was PCR‐amplified as two fragments using the primer pairs listed in Table S2 and the plasmid pAO4‐13 (de Ruiter‐Jacobs et al., 1989) as template. Split marker fragments with the *AOpyrG* selection marker were created by fusion PCR and used to transform the strain MA169.4 (Carvalho, Arentshorst, Kwon, Meyer, & Ram, 2010), resulting in the strain SO1.1. Proper deletion of *gaaR* was confirmed by diagnostic PCR (data not shown) and via Southern blot analysis.

### 2.6 Construction of strains expressing *gaaR* ^*W361R*^, *gaaR* ^*W361R*^ *‐eGFP*, and *gaaR‐eGFP* at the endogenous *gaaR* locus

To construct plasmid pSO1.2, the P*gaaR*‐*gaaR* ^*W361R*^‐T*gaaR* allele was amplified by PCR using the primers gaaRP5f and gaaRP6r (Table S2) with JN103.1 genomic DNA as template and ligated into pJET1.2/blunt cloning vector (Thermo Fisher Scientific). The resulting plasmid pSO1.2 was amplified in *E. coli*. pSO2.1 (containing the P*gaaR*‐*gaaR* ^*W361R*^‐*eGFP*‐T*gaaR* construct) was created by digesting the plasmids pSO1.2 and pEA9 (containing the P*gaaR*‐*gaaR*‐*eGFP*‐T*gaaR* construct) with the restriction enzymes *Bcu*I and *Bam*HI, and by ligating the 2408‐bp *Bcu*I‐*Bam*HI fragment from pSO1.2 (containing the *gaaR* ^*W361R*^ mutation) with the *Bcu*I and *Bam*HI cut opened pEA9. pSO1.2 and pSO2.1 were sequenced to ensure no PCR errors have occurred and proper ligation and orientation of the fragments.

Repair fragments for CRISPR‐Cas9 mediated targeted integration (see below) were obtained as follows: The P*gaaR*‐*gaaR* ^*W361R*^‐T*gaaR* construct containing the 964‐bp P*gaaR* and 992‐bp T*gaaR* was excised from pSO1.2 using the restriction enzymes *Not*I and *Xba*I. The P*gaaR*‐*gaaR*‐*eGFP*‐T*gaaR* and P*gaaR*‐*gaaR* ^*W361R*^‐*eGFP*‐T*gaaR* constructs containing the 731‐bp P*gaaR* and 419‐bp T*gaaR* were excised from pEA9 and pSO2.1, respectively, using the restriction enzyme *Bgl*II.

The strains SO2.1 (*gaaR* ^*W361R*^), EA31.1 (*gaaR‐eGFP*), and EA32.1 (*gaaR* ^*W361R*^ *‐eGFP*) were created using the CRISPR‐Cas9 technique (Nødvig, Nielsen, Kogle, & Mortensen, 2015; manuscript in preparation Song et al., 2018). A guide RNA protospacer sequence GTGGATACGTACTCCTTTTA targeting the *AOpyrG* gene was designed using E‐CRISP (Heigwer, Kerr, & Boutros, 2014).

To create SO2.1, the DNA template for the in vitro synthesis of the guide RNA via the T7 promoter was amplified by PCR using the primers sgRNAP1 and OTL19 (Table S2), and the plasmid p426‐SNR52p‐gRNA.CAN1.Y‐SUP4t (Addgene, USA) (DiCarlo et al., 2013) as template. The PCR consisted of the following reactions: Initial denaturation at 98°C for 30 s; 30 cycles of denaturation at 98°C for 5 s, annealing at 77°C for 5 s, extension at 72°C for 2 s; and final extension at 72°C for 30 s. The amplified DNA template was transcribed in vitro using the MEGAscript T7 Kit (Thermo Fisher Scientific). P*gaaR*‐*gaaR* ^*W361R*^‐T*gaaR* repair fragment was co‐transformed into strain SO1.1 (Δ*gaaR::AOpyrG*) together with the in vitro transcribed guide RNA and the plasmid pFC332 containing the *cas9* gene and the hygromycin selection marker (Nødvig et al., 2015), yielding to SO2.1. Transformants were selected on transformation plates containing hygromycin and uridine, and purified twice on MM containing GA, 5′‐fluoroorotic acid, uridine, and hygromycin. pFC332 was cured by further purifying transformants twice without hygromycin selection on MM containing glucose and uridine. Transformants were not able to grow on MM containing glucose, uridine, and hygromycin after the first round of purification, indicating that pFC332 was successfully cured.

The DNA template for the in vivo synthesis of the guide RNA via the RNA polymerase III promoter was amplified in two parts by PCR using the primers Fw_LIC2 and Rev_P1, and Fw_P1 and Rev_LIC2 (Table S2) using the plasmid ANEp8_Cas9‐gRNAalbA (manuscript in preparation Song et al., 2018) as template. The two PCR products were combined by fusion PCR using the primers Fw_LIC2 and Rev_LIC2. The fusion PCR product was amplified by PCR using the primers for_pTE1 and rev_pTE1 to introduce *Pac*I restriction sites at both ends, digested with *Pac*I, and ligated into *Pac*I cut opened pFC332, yielding to plasmid pTE1. pTE1 was co‐transformed together with P*gaaR*‐*gaaR*‐*eGFP*‐T*gaaR* or P*gaaR*‐*gaaR* ^*W361R*^‐*eGFP*‐T*gaaR* repair fragments into the strain SO1.1 (Δ*gaaR::AOpyrG*) to create EA31.1 or EA32.1, respectively. Transformants were selected on transformation plates containing hygromycin and uridine, and purified twice on MM containing GA, 5′‐fluoroorotic acid and uridine. Transformants were not able to grow on MM containing GA, 5′‐fluoroorotic acid, uridine, and hygromycin after the first round of purification, indicating that pTE1 was successfully cured.

The *gaaR* locus in SO2.1 and EA31.1 was amplified using the primers gaaRP7f and gaaRP8r, and the one in EA32.1 using the primers PgaaR_seq_P2 and TgaaR_seq_P2, and ligated into pJET1.2/blunt cloning vector (Thermo Fisher Scientific). The resulting plasmids were amplified in *E. coli* and sequenced using *gaaR* sequencing primers to confirm the presence of the mutated *gaaR* allele in SO2.1 and EA32.1, and the wild‐type *gaaR* allele in EA31.1. Integration of the repair fragments into the endogenous *gaaR* locus in SO2.1, EA31.1, and EA32.1 was confirmed via Southern blot analysis (Figure S3). SO2.1, EA31.1, and EA32.1 were made uridine prototroph by transforming with the plasmid pAB4.1 (van Hartingsveldt, Mattern, van Zeijl, Pouwels, & van den Hondel, 1987), yielding to strains EA34.1, EA36.1, and EA39.1, respectively.

### 2.7 Enzymatic analysis

Supernatants from shake flask cultures were obtained by filtration through glass microfiber filters (Whatman, Buckinghamshire, UK), and the filtrate was stored at −80°C. PGA plate assays were performed as described by Niu et al. (2017). Twenty‐five microliters of supernatant from each culture were spotted on plates containing 0.2% PGA, and plates were incubated at 37°C for 24 hr before staining with 0.1% Congo Red (Sigma‐Aldrich, Zwijndrecht) solution.

### 2.8 Microscopy

The cover slips with adherent germlings were placed upside down on glass slides. The GFP fluorescence was excited using 488 nm laser line in a Zeiss Observer confocal laser scanning microscope (Zeiss, Jena, Germany). The images were analyzed with the ImageJ software (Abramoff, Magalhaes, & Ram, 2004). On each image, the exact same brightness and contrast adjustments were applied.

## 3 RESULTS

### 3.1 Genetic characterization of *A. niger* mutants showing constitutive production of pectinases

In a previous study, we isolated 64 mutants showing constitutive production of pectinases (Niu et al., 2017). Genome sequencing of five mutants showed that all five mutants harbor a mutation in a putative repressor protein that represses the expression of pectinases under non‐inducing conditions (Niu et al., 2017). We sequenced the *gaaX* gene in the remaining 59 mutants showing constitutive production of pectinases (Table S3, Figure S1). In total, 28 and nine mutants were found to carry nonsense and frameshift mutations, respectively. Twenty‐three mutants were found to have missense mutations in the *gaaX* gene. These missense mutations were found throughout *gaaX*, indicating the importance of the different domains of GaaX for proper functioning. The nonsense or frameshift mutation closest to the C‐terminus was the frameshift mutation V653G, indicating the importance of the last 45 amino acid residues for a functional GaaX, where the missense mutations L676P and V684K were also found (Figure S1). Four mutants did not carry any mutations in *gaaX*. Sequencing the *gaaR* gene in these mutants resulted in the identification of a mutation in *gaaR* in one of the mutant strains (JN103.1).

**Figure 1:**
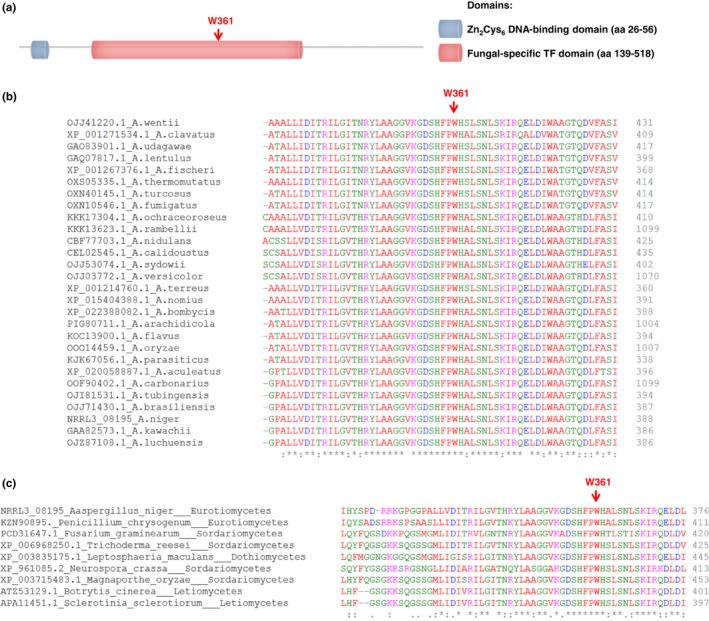
Schematic representation of the domains present in GaaR (a) and conservation of GaaR^W^ ^361^ in *Aspergillus* species (b) and other Ascomycetes (c). Domains in GaaR (NRRL3_08195) were identified using NCBI's CDD (Marchler‐Bauer et al., 2015). Protein sequences homologous to the *Aspergillus niger* GaaR were retrieved using the blastp algorithm from NCBI (Altschul, Gish, Miller, Myers, & Lipman, 1990) against the nonredundant protein sequences database and were aligned using Clustal Omega (Sievers et al., 2011)

The UV mutant P*pgaX*‐*amdS ∆creA gaaR* ^*W361R*‐UV^ (JN103.1) carries a missense mutation (W 361 to R) caused by codon change from TGG to CGG at a distance of 1285 bp from the start codon of *gaaR*. The tryptophan361 residue lies in the fungal‐specific transcription factor domain (cd12148) that spans the residues 139‐518 in the 740 amino acid‐long GaaR (Figure 1a), and is 100% conserved in the homologous GaaR sequences present in *Aspergillus* species (Figure 1b) and in other Ascomycetes analyzed belonging to the Pezizomycotina subdivision (Figure 1c).

### 3.2 Analyses of pectinase production in *gaaR* ^*W361R*^

**Figure 2:**
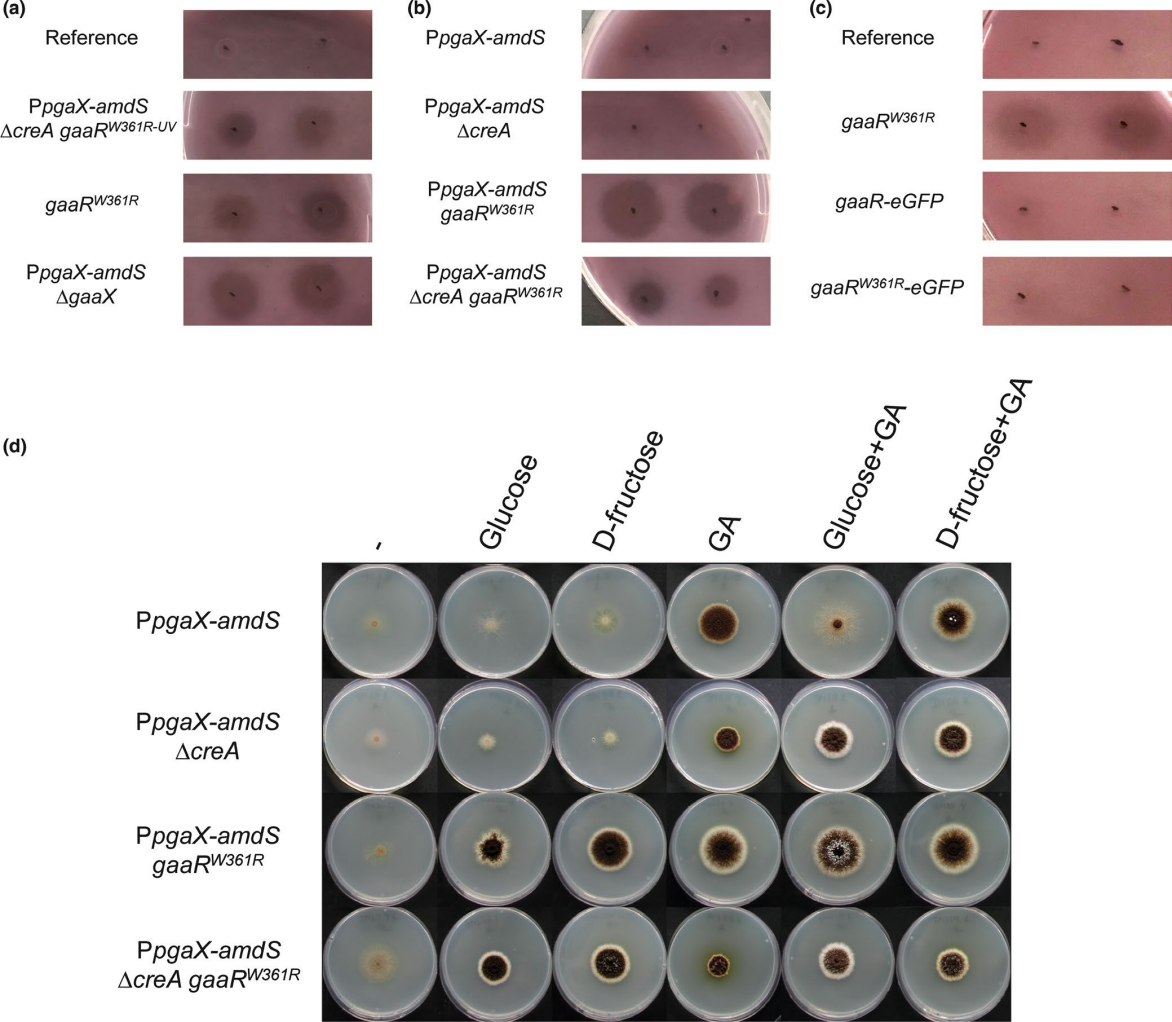
Effect of the W361R mutation in *gaaR* on the expression of pectinase genes. (a–c) PGA plate assay. Strains were grown in MM containing 50 mM D‐fructose for 36 hr, and culture supernatants were spotted PGA plates. Enzymatic activities in the supernatants from duplicate cultures are shown: (a) the reference (MA234.1), P*pgaX*‐*amdS ∆creA gaa* *R*^*W*^ ^*361R*‐^ ^UV^ (JN103.1), *gaa* *R*^*W*^ ^*361R*^ (EA34.1), P*pgaX*‐*amdS ∆gaaX* (JN123.1); (b) P*pgaX*‐*amdS* (JC1.5), P*pgaX*‐*amdS ∆creA* (JN29.2), P*pgaX*‐*amdS gaa* *R*^*W*^ ^*361R*^ (JN130.4), P*pgaX*‐*amdS ∆creA gaa* *R*^*W*^ ^*361R*^ (JN129.1); and (c) reference (MA234.1), *gaa* *R*^*W*^ ^*361R*^ (EA34.1), *gaaR‐eGFP* (EA36.1), and *gaa* *R*^*W*^ ^*361R*^ *‐eGFP* (EA39.1). (d) Growth phenotype of the P*pgaX*‐*amdS* (JC1.5), P*pgaX*‐*amdS ∆creA* (JN29.2), P*pgaX*‐*amdS gaa* *R*^*W*^ ^*361R*^ (JN130.4), and P*pgaX*‐*amdS ∆creA gaa* *R*^*W*^ ^*361R*^ (JN129.1) strains after 7 days at 30°C on solid MM containing no carbon source (−); 50 mM glucose, D‐fructose or GA as the sole carbon source; or 50 mM glucose or D‐fructose together with 50 mM GA. All plates contain 10 mM acetamide as the sole nitrogen source

To show that the W361R mutation in GaaR is responsible for the constitutive production of pectinases, the GaaR gene containing the W361R mutation was introduced via a CRISPR/Cas9 approach into the endogenous *gaaR* locus in SO1.1, a strain carrying Δ*gaaR::AOpyrG*. Southern blot analysis indicated that the P*gaaR*‐*gaaR* ^*W361R*^‐T*gaaR* construct was successfully integrated into the endogenous *gaaR* locus in the genome of strain SO2.1 (Figure S3), thereby replacing the *A. oryzae pyrG* marker which was used for deleting *gaaR*. Growth of SO2.1 (*gaaR* ^*W361R*^) was analyzed on different monomeric and polymeric carbon sources (Figure S2). Strain SO1.1 (Δ*gaaR*) displayed strongly impaired growth on GA, PGA, and AP, as shown previously (Alazi et al., 2016). Introduction of *gaaR* ^*W361R*^ gene (strain SO2.1) resulted in a restoration of growth on GA‐containing carbon sources, indicating the presence of a functional GaaR. The presence of the *gaaR* ^*W361R*^ allele did not affect growth on any other carbon sources tested. The pectinase production capacity of *gaaR* ^*W361R*^ (EA34.1), was assessed via PGA plate assay (Figure 2a). Strains were grown in liquid medium containing D‐fructose as a non‐inducing carbon source, and the culture supernatants were spotted on PGA plates. Comparison of hydrolysis zones on PGA plates showed that *gaaR* ^*W361R*^ (EA34.1) constitutively produces pectinases similar to the UV mutant P*pgaX*‐*amdS ∆creA gaaR* ^*W361R*‐UV^ (JN103.1) (Figure 2a). This indicates that the W361R mutation in *gaaR*, and no other additional mutation(s) induced by UV mutagenesis in P*pgaX*‐*amdS ∆creA gaaR* ^*W361R*‐UV^ (JN103.1), is responsible for the constitutive production of pectinases.

The effect of the W361R mutation in *gaaR* on the expression of pectinase genes was also analyzed using the promoter‐reporter strains expressing the *amdS* gene via the *pgaX* promoter. The *gaaR* ^*W361R*^ gene was transformed into the P*pgaX*‐*amdS* (JC1.5) and P*pgaX*‐*amdS ∆creA* (JN29.2) strains. Transformants were selected on medium containing a non‐inducing carbon source and acetamide as the nitrogen source, and only transformants in which the *gaaR* ^*W361R*^ was integrated were expected to grow on the transformation plate because of constitutive activation of the P*pgaX*‐*amdS* reporter. Southern blot analysis and sequencing of the *gaaR* locus showed that the endogenous *gaaR* gene was replaced with *gaaR* ^*W361R*^ in the resulting strains P*pgaX*‐*amdS gaaR* ^*W361R*^ (JN130.4) and P*pgaX*‐*amdS ∆creA gaaR* ^*W361R*^ (JN129.1), respectively (Figure S3). Growth of P*pgaX*‐*amdS gaaR* ^*W361R*^ (JN130.4) and P*pgaX*‐*amdS ∆creA gaaR* ^*W361R*^ (JN129.1) was similar to the growth of their parental strains on MM containing glucose, D‐fructose, GA, PGA, or AP (Figure S2).

The PGA plate assay showed that the parental strains P*pgaX*‐*amdS* (JC1.5) and P*pgaX*‐*amdS ∆creA* (JN29.2) did not produce any polygalacturonases in D‐fructose, while P*pgaX*‐*amdS gaaR* ^*W361R*^ (JN130.4) and P*pgaX*‐*amdS ∆creA gaaR* ^*W361R*^ (JN129.1) showed polygalacturonase activity in their culture supernatants (Figure 2b). The decreased polygalacturonase activity in the culture supernatant of P*pgaX*‐*amdS ∆creA gaaR* ^*W361R*^ (JN129.1) compared to that of P*pgaX*‐*amdS gaaR* ^*W361R*^ (JN130.4) might be caused by the growth defect due to *creA* deletion.

The expression of *pgaX* was previously shown to be induced only in the presence of an inducing carbon source, such as GA (Niu et al., 2015). Radial growth assays on plates containing acetamide showed that P*pgaX*‐*amdS gaaR* ^*W361R*^ (JN130.4) and P*pgaX*‐*amdS ∆creA gaaR* ^*W361R*^ (JN129.1) can grow on glucose or D‐fructose, while their parental strains cannot (Figure 2d). This indicates that GaaR^W361R^ can activate the expression of *amdS* via the *pgaX* promoter in an inducer‐independent way, confirming the results obtained by PGA plate assays.

The expression of *pgaX* is known to be repressed strongly by glucose and mildly by D‐fructose in a CreA‐dependent way (Alazi et al., 2018; Niu et al., 2015). These previous results were confirmed as shown in Figure 2d, where growth of the promoter‐reporter strains on plates containing acetamide and GA decreased when glucose was added to the growth media, and a short delay in growth and sporulation was observed when fructose was added as a repressing sugar. The repressing effect of glucose or fructose was lost upon *creA* deletion, indicating that CreA is required for the repressing effect of glucose and fructose. To examine whether the CreA‐mediated carbon catabolite repression was dominant over constitutive activation of GaaR, the growth the P*pgaX*‐*amdS gaaR* ^*W361R*^ reporter strain was analyzed under non‐inducing/repressing conditions (Figure 2d). The P*pgaX*‐*amdS gaaR* ^*W361R*^ (JN130.4) could grow both on glucose and D‐fructose as the sole carbon source indicating constitutive expression even under carbon catabolite repressing conditions (Figure 2d). The colony growth on glucose was irregular and reduced compared to the growth on fructose, indicating that constitutive activation of GaaR was not fully dominant over CreA‐mediated repression. Deletion of *creA* in the P*pgaX*‐*amdS gaaR* ^*W361R*^ reporter strain alleviated glucose repression indicating that CreA‐mediated repression on *pgaX* plays an important role even in the presence of the constitutively active GaaR^W361R^.

### 3.3 Subcellular localization of *GaaR‐eGFP* and *GaaR* ^*W361R*^ *‐eGFP*

We previously showed that *eGFP*‐*gaaR* (EA19.2), a strain expressing the N‐terminally eGFP‐tagged *gaaR*, exhibits slightly reduced growth on GA compared to the reference strain (Alazi et al., 2018), indicating that N‐terminal eGFP‐tagging might affect the function of GaaR. To check whether C‐terminal eGFP‐tagging affects GaaR functionality, strains expressing C‐terminally eGFP‐tagged GaaR, with or without a linker between GaaR and eGFP, were created in a *∆gaaR* background. While *∆gaaR* (JN35.1) could not grow on GA, growth on GA was restored in both *gaaR‐eGFP* (EA29.14) and *gaaR‐(GA)*_*4*_ *‐eGFP* (EA30.6) (Figure S4). This indicates that C‐terminal eGFP‐tagging does not affect the function of GaaR, independently of the presence of a linker between GaaR and eGFP.

To analyse the subcellular localization of GaaR‐eGFP and GaaR^W361^‐eGFP, strains that express C‐terminally eGFP‐tagged *gaaR* or *gaaR* ^*W361R*^ from the endogenous *gaaR* locus were created, and integration of the P*gaaR*‐*gaaR*‐*eGFP*‐T*gaaR* or P*gaaR*‐*gaaR* ^*W361R*^‐*eGFP*‐T*gaaR* constructs into the endogenous *gaaR* locus was verified by Southern blot analysis (Figure S3). Growth of *gaaR‐eGFP* (EA31.1) and *gaaR* ^*W361R*^ *‐eGFP* (EA32.1) was complemented on GA and GA‐containing carbon sources (Figure S2), confirming previous results that GaaR‐eGFP is functional under inducing conditions.

The ability of *gaaR‐eGFP* (EA36.1) and *gaaR* ^*W361R*^ *‐eGFP* (EA39.1) to constitutively produce pectinases was assessed via a PGA plate assay and compared to that of *gaaR* ^*W361R*^ (EA34.1) (Figure 2c). No polygalacturonase activity was observed in the culture supernatant of the reference (MA234.1) strain or *gaaR‐eGFP* (EA36.1) after growth in D‐fructose, indicating that C‐terminal eGFP‐tagging does not affect the inactive state of GaaR under non‐inducing conditions, similar to N‐terminal eGFP‐tagging (Alazi et al., 2018). While the *gaaR* ^*W361R*^ (EA34.1) strain expressing the constitutively active *gaaR* ^*W361R*^ produced pectinases in D‐fructose, the *gaaR* ^*W361R*^ *‐eGFP* (EA39.1) strain expressing *gaaR* ^*W361R*^ *‐eGFP* did not (Figure 2c). This indicates that GFP‐tagging of the C‐terminus of GaaR^W361R^ interferes with constitutive function of the protein.

**Figure 3:**
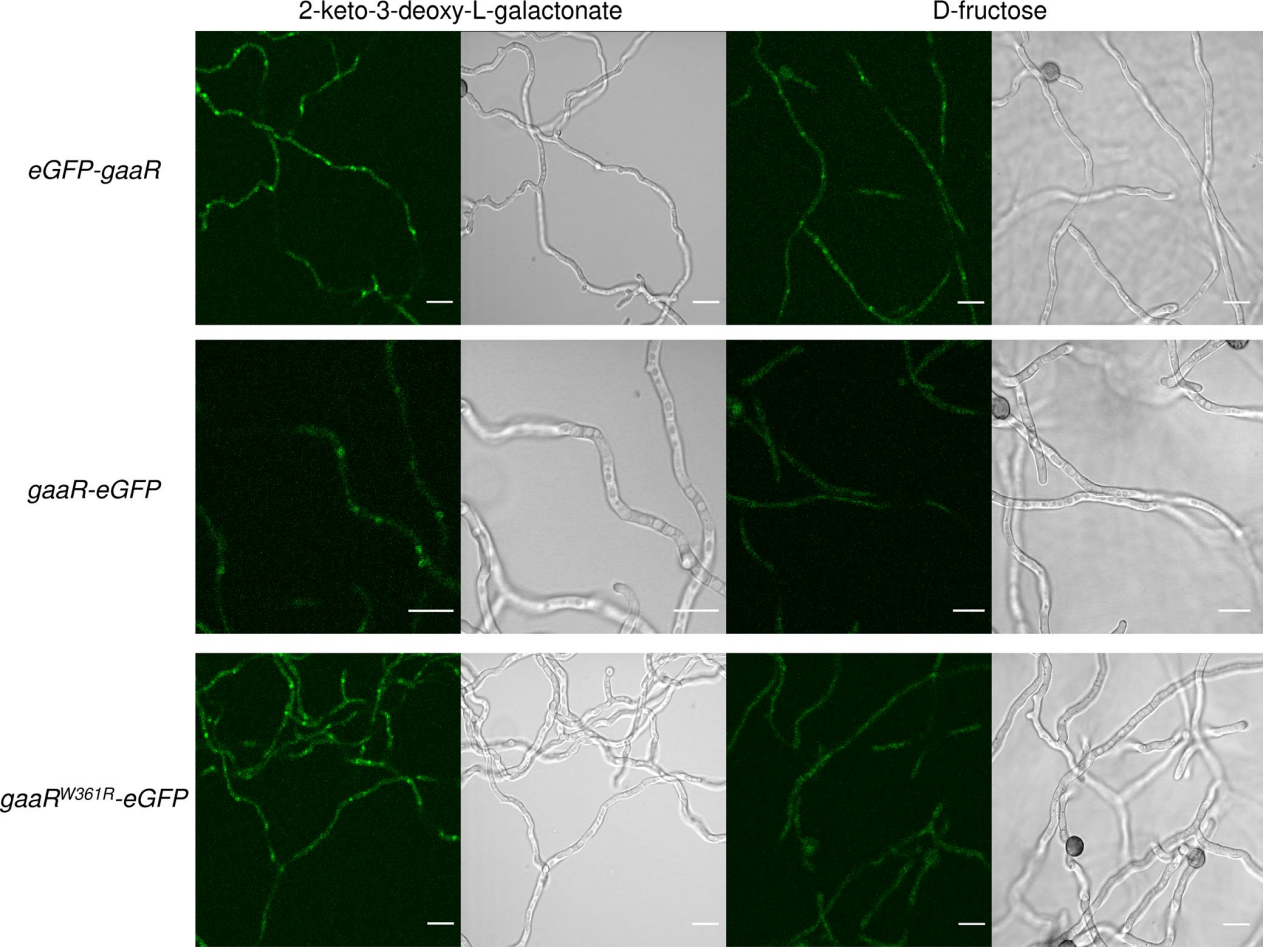
Nuclear and cytoplasmic fluorescence intensities of the eGFP‐tagged GaaR and GaaR^W^ ^361R^ proteins. The *eGFP*‐*gaaR* (EA19.2), *gaaR‐eGFP* (EA36.1) and *gaa* *R*^*W*^ ^*361R*^ *‐eGFP* (EA39.1) strains were grown in MM containing 10 mM D‐fructose or 2‐keto‐3‐deoxy‐L‐galactonate for 21 hr. Example micrographs representing each condition are shown. Scale bar: 10 μm

The subcellular localization of the C‐terminally eGFP‐tagged GaaR and GaaR^W361R^ in *gaaR‐eGFP* (EA36.1) and *gaaR* ^*W361R*^ *‐eGFP* (EA39.1) grown under inducing (i.e., on 2‐keto‐3‐deoxy‐L‐galactonate) or non‐inducing (i.e., on D‐fructose) conditions was analyzed using confocal laser scanning microscopy (Figure 3). Beforehand, the potency of 2‐keto‐3‐deoxy‐L‐galactonate, the physiological inducer (Alazi et al., 2017), to induce the expression of the GA‐responsive genes was tested using the promoter–reporter strain P*pgaX*‐*amdS ∆creA* (JN29.2) and was compared to that of GA (Figure S5). Addition of 100 nM 2‐keto‐3‐deoxy‐L‐galactonate to the growth media resulted in a major increase in growth, whereas addition of a 100 nM GA was not sufficient to induce the growth of P*pgaX*‐*amdS ∆creA* (JN29.2), indicating that 2‐keto‐3‐deoxy‐L‐galactonate is able to induce *pgaX* expression and that 2‐keto‐3‐deoxy‐L‐galactonate is more potent than GA in inducing the expression.

We previously showed that the partially functional N‐terminally eGFP‐tagged GaaR in *eGFP*‐*gaaR* (EA19.2) is localized mainly in the nucleus under both inducing (i.e., on GA) and non‐inducing (i.e., on D‐fructose) conditions (Alazi et al., 2018). Here, we repeated this experiment using 2‐keto‐3‐deoxy‐L‐galactonate as an inducer instead of GA and confirmed a visual signal of eGFP‐GaaR in the nucleus after induction by 2‐keto‐3‐deoxy‐L‐galactonate or on the non‐inducing carbon source D‐fructose (Figure 3). C‐terminally eGFP‐tagged GaaR and GaaR^W361R^ were also visualized mostly in the nucleus after growth in 2‐keto‐3‐deoxy‐L‐galactonate. Surprisingly, this was not observed after growth in D‐fructose. This result indicates that on D‐fructose (under non‐inducing and mildly repressing conditions) nuclear accumulation of C‐terminally eGFP‐tagged GaaR is restrained and suggests that the C‐terminal tagging of GaaR with eGFP affects is localization under non‐inducing conditions. The inability of the GaaR‐eGFP protein to localize in the nucleus under non‐inducing conditions is most likely also the reason why the GaaR^W361R^‐eGFP protein does not exert its constitutive activity.

## 4 DISCUSSION

We previously isolated via a forward genetic screen *A. niger* strains displaying constitutive expression of pectinases (Niu et al., 2017). The majority of the mutants in the collection (60 out of 64 strains) was found to contain mutations in GaaX and are probably all loss‐of‐function mutations as deletion of *gaaX* leads to the same phenotype (Niu et al., 2017). From the four mutants that did not carry mutations in *gaaX*, one mutant was found to possess a missense mutation leading to an amino acid change from tryptophan to arginine at position 361 in GaaR. The nature of the mutations in the three UV mutants with no mutations in *gaaX* or *gaaR* remains to be discovered. Introducing the GaaR^W361R^ mutation in the wild‐type genetic background and subsequent phenotypic analyses allowed us to conclude that this mutation results in constitutive activation of GaaR and therefore constitutive production of pectinases under non‐inducing conditions.

To our knowledge, XlnR/Xyr1 is the only transcription factor involved in plant biomass degradation in which missense mutations leading to constitutive expression of enzymes are described. The two mutations are the XlnR^V756F^ mutation in *A. niger*, and the Xyr1^A824V^ mutation in *Trichoderma reesei* and orthologous mutations in *N. crassa* and *Penicilium oxalicum*, resulting in constitutive production of xylanases (Craig, Coradetti, Starr, & Glass, 2015; Derntl et al., 2013; Gao et al., 2017; Hasper, 2004; Hasper, Trindade, van der Veen, van Ooyen, & de Graaff, 2004). In protein sequence alignment, these two mutations are only three amino acids apart from each other (Figure S6). Whereas the GaaR^W361R^ mutation is located roughly half way of the 740 amino acid‐long GaaR protein, the XlnR^V756F^ mutation is located closer to the C‐terminal in the 875 amino acid‐long XlnR protein. The GaaR^W361R^ mutation is predicted to be in the fungal‐specific transcription factor domain (cd12148) which comprises amino acids 139–518 in GaaR. The cd12148 domain comprises amino acids 341–653 in *A. niger* XlnR and 368–717 in *T. reesei* Xyr1 (NCBI's CDD (Marchler‐Bauer et al., 2015)), and the constitutive mutations in XlnR/Xyr1 are therefore outside of the predicted fungal‐specific transcription factor domain in the C‐terminal parts of XlnR and Xyr1, which has been suggested to be the activation domain. The XlnR/Xyr1 constitutive mutations are not located in a defined protein domain family, but the C‐terminal parts of XlnR and Xyr1, including the amino acids V756 and the A824, respectively, have been suggested to be the activator domain (Derntl et al., 2013; Hasper et al., 2004). Alignment of protein sequences of XlnR and GaaR from *A. niger*,* A nidulans*,* Aspergillus fumigatus* and *A. oryzae* and Xyr1 of *T. reesei* using Clustal omega (Sievers et al., 2011) showed low sequence similarity in the amino acid residues surrounding the GaaR^W361^ residue between GaaR and XlnR/Xyr1 (Figure S6). Whereas the W361 residue is fully conserved in GaaR in all four *Aspergilli*, only *A. niger, A. oryzae,* and *T. reesei* had a W residue positioned at the aligned protein sequence of XlnR/Xyr1 (Figure S6). Of note is that sequence similarity surrounding this region of XlnR/Xyr1 is weak, making it possible that this conservation has no biological relevance. To test its biological relevance, it will be of interest to examine whether mutations in XlnR of *A. niger* at position W639 would also result in a constitutive phenotype. Protein sequence alignment revealed that the V756 and A824 residues are fully conserved in the XlnR/Xyr1 proteins of the four *Aspergilli* and *T. reesei*, but not strictly conserved between XlnR/Xyr1 and GaaR (Figure S6). Because of the low level of conservation between the regions and amino acids that result in constitutive activation of the respective transcription factors, it is reasonable to suggest that the mechanisms leading to the activation of GaaR and XlnR/Xyr1 are different. We identified in the *A. niger* genome four transcription factor/repressor modules (Niu et al., 2017). For two modules, we know that they are involved in either GA or quinic acid utilization (Niu et al., 2017; Ram et al., unpublished results). Analysis of the knockouts for the two remaining genes encoding the repressors, and subsequent phenotypic and transcriptomic analysis has shown that the two repressors are not involved in the expression of genes encoding xylanases (Alazi and Ram, unpublished results). Therefore, the activity of XlnR is likely not controlled by a GaaX‐like repressor protein.

The activity of GaaR is proposed to be inhibited by GaaX under non‐inducing conditions via protein–protein interactions (Niu et al., 2017). Pectinase genes are expressed under inducing conditions (i.e., in the presence of inducer), or under non‐inducing conditions (i.e., in the absence of inducer) when *gaaX* is deleted or *gaaR* is overexpressed (Alazi et al., 2018; Martens‐Uzunova & Schaap, 2008; Niu et al., 2017). These findings indicate that GA‐responsive gene expression only requires the presence of active GaaR relieved/escaped from GaaX inhibition but does not require the presence of the inducer. This suggests that the inducer binds to GaaX under inducing conditions resulting in a GaaX‐unbound and active form of GaaR. Therefore, we propose that W361R in GaaR disrupts the interaction between GaaX and GaaR under non‐inducing conditions. Both *PpgaX‐amdS gaaR* ^*W361R*^ and *PpgaX‐amdS ∆gaaX* mutants were found to produce pectinases under non‐inducing conditions (Figure 2a and b). The qualitative phenotypic similarity of these strains resulting from the W361R mutation in GaaR or the absence of GaaX, respectively, suggests that the GaaR‐GaaX interaction is disturbed in the GaaR^W361R^ mutant. In future experiments, the proposed protein–protein interaction of GaaR and GaaX and mutant variants thereof will be studied further in detail. Initial attempts to express GaaR and GaaX in *E. coli* to perform interaction studies have been unsuccessful and will be subject for further research. In this respect, it would also be of interest to isolate and identify additional mutations in GaaR that lead to constitutive expression of pectinases to identify regions in GaaR that are necessary for the proposed interaction with GaaX.

GaaX of *A. niger*, QutR of *A. nidulans,* and qa‐1S of *N. crassa* are all multidomain repressor proteins with sequence similarity to each other and to the three C‐terminal domains of AROM, a large pentafunctional protein involved in the shikimate pathway. This suggests that these repressors share a common evolutionary origin. The last three domains of the AROM protein encode the shikimate kinase, 3‐dehydroquinate dehydratase, and shikimate dehydrogenase enzymes (Lamb et al., 1996). The sequence similarity of the repressor domains to AROM is low (between 20% and 30% identity), suggesting that the enzymatic functions that are present in AROM are lost in the repressors. Loss‐of‐function mutations (nonsense and missense mutations) were found throughout all three domains of GaaX (shikimate kinase, 3‐dehydroquinate dehydratase, and shikimate dehydrogenase domains) (Figure S1). The 22 missense mutations could especially be valuable for structure/function relationships to predict which domains exert different function as they might affect the proposed protein–protein interaction between GaaX and GaaR. It is however difficult to predict whether a missense mutation affects the overall secondary/tertiary structure of the protein or whether a mutation affects the interaction without affecting its structure. We identified a hot spot for mutations in the region 190–194 in which four mutants were found carrying mutations in three amino acid positions. This region could either be important for GaaR–GaaX interaction or could be important for the structure of GaaX. Alignment of protein sequences homologous to GaaX present in 18 Pezizomycetes species using Clustal omega (Sievers et al., 2011) revealed that these four mutations were found in three residues that are highly conserved (Figure S7). Several other missense mutations were found in conserved residues and are indicated in Figures S1 and S7. Relatively more missense mutations were found in the shikimate dehydrogenase domain (one mutation per 24 amino acids) (Figure S1) compared to the shikimate kinase (one mutation per 76 amino acids), or 3‐dehydroquinate dehydratase domains (one mutation per 37 amino acids), indicating that the shikimate dehydrogenase domain might be more important in relation to binding to GaaR than the other domains.

In *N. crassa*, dehydroquinic acid is suggested to be the physiological inducer of the quinic acid catabolic pathway genes, as mutants that accumulate or cannot produce dehydroquinic acid showed induced or diminished expression of these genes, respectively (Giles, Partridge, Ahmed, & Case, 1967). On the other hand, Huiet and Giles identified two missense mutations in non‐inducible *N. crassa* strains in the Qa‐1S domain showing homology with the AROM shikimate dehydrogenase, indicating that this domain might interact with the inducer (Huiet & Giles, 1986). Hawkins, Lamb, Moore, Charles, and Roberts (1993) have proposed that the QutR domain homologous to the AROM 3‐dehydroquinate dehydratase can recognize and bind to dehydroquinic acid produced from quinic acid, but does not react on it enzymatically in *A. nidulans*. For *A. niger*, accumulation of 2‐keto‐3‐deoxy‐L‐galactonate in Δ*gaaC* results in hyper‐induction of GA‐responsive genes and the inducer 2‐keto‐3‐deoxy‐L‐galactonate is likely to interact with the GaaX repressor protein (Alazi et al., 2017). The GA catabolic pathway enzyme GaaC is an aldolase and converts 2‐keto‐3‐deoxy‐L‐galactonate to pyruvate and L‐glyceraldehyde. Both the aldolase domain of GaaC and the 3‐dehydroquinate dehydratase domain of GaaX belong to the same Aldolase Class I_superfamily (NCBI's CDD Marchler‐Bauer et al., 2015). These findings suggest that 2‐keto‐3‐deoxy‐L‐galactonate could bind to the GaaX domain homologous to the AROM 3‐dehydroquinate dehydratase. However, direct protein‐metabolite interaction studies are required to determine which part (or parts) of the repressor protein interacts with the inducer.

N‐terminally eGFP‐tagged GaaR was previously shown to mainly localize in the nucleus under both inducing and non‐inducing conditions but was unable to fully complement the growth of *∆gaaR* when grown on inducing carbon sources (Alazi et al., 2018). C‐terminally eGFP‐tagged GaaR/GaaR^W361R^ accumulated in the nucleus under inducing conditions and complemented the growth of *∆gaaR* when grown on inducing carbon sources. Based on these observations, we conclude that C‐terminally eGFP‐tagged GaaR/GaaR^W361R^ is functional under inducing conditions. However, prominent nuclear eGFP‐fluorescence signal was not observed in the strains producing the C‐terminally eGFP‐tagged GaaR/GaaR^W361R^ under non‐inducing conditions. This result indicates that C‐terminal eGFP‐tagging restrained nuclear accumulation of GaaR/GaaR^W361R^ under non‐inducing conditions, explaining the inability of GaaR^W361R^‐eGFP to constitutively activate the production of pectinases, as GaaR^W361R^ can. In *B. cinerea*, expression of the C‐terminally GFP‐tagged *BcgaaR* via the strong *oliC* promoter resulted in higher nuclear GFP fluorescence intensity under both inducing and non‐inducing conditions (Zhang et al., 2016). This indicates that nuclear accumulation of overexpressed and wild‐type BcGaaR is not restrained by C‐terminal GFP‐tagging under non‐inducing conditions, unlike the GaaR‐eGFP/GaaR^W361^‐eGFP expressed from its endogenous promoter.

Constitutive production of plant biomass degrading enzymes like pectinases is interesting for the industrial production of these enzymes because it allows the production independently of the feedstock. By understanding the transcriptional regulatory mechanism of pectinases, several approaches have been conducted leading to inducer independent and abundant production of pectinases, such as deletion of GaaX (Niu et al., 2017), overexpression of GaaR from a constitutive promoter (Alazi et al., 2018), or accumulation of the inducer (Alazi et al., 2017). Here, we show that a mutation in GaaR can also lead to constitutive production of pectinases. Combination of these approaches can be considered to further boost pectinase production in *A. niger*. The conservation of GaaR and GaaX in other fungi also allows improved pectinase production in other industrially important filamentous fungi.

## CONFLICT OF INTEREST

The authors declare no conflict of interest.

## AUTHORS CONTRIBUTION

EA, JN, SBO, MA, and TTMP performed the experiments, EA, AT, and AFJR designed the experiments and wrote the manuscript with input of all the authors.

## Data Availability

Strains, plasmids, and primers in this study are stored at the University of Leiden and are available via the corresponding author.
