# Supplementary material for: W361R mutation in GaaR, the regulator of D‐galacturonic acid‐responsive genes, leads to constitutive production of pectinases in Aspergillus niger
Source: Microbiologyopen. 2018 Oct 8;8(5):e00732. doi: 10.1002/mbo3.732 (PMC6528562; doi:10.1002/mbo3.732)
Supplement: Supplementary file 1 [file MBO3-8-e00732-s001.pdf]

MicrobiologyOpen

W361R mutation in GaaR, the regulator of D-galacturonic acid responsive genes, leads to constitutive production of pectinases in *Aspergillus niger*

Ebru Alazi[1], Jing Niu[1], Simon B. Otto[1], Mark Arentshorst[1], Thi T. M. Pham[2], Adrian Tsang[2] and Arthur F.J. Ram[1]

[1] Molecular Microbiology and Biotechnology, Institute of Biology Leiden, Leiden University, The Netherlands

[2] Centre for Structural and Functional Genomics, Concordia University, Canada

a.f.j.ram@biology.leidenuniv.nl

Table S1. Strains used in this study.

Strain	Accession Number	Genotype	Description	Reference
MA169.4	FGSC A1279	*cspA1, pyrG^{378}, kusA::DR-amdS-DR*	Transiently silenced *kusA* in AB4.1	Carvalho *et al.*, 2010
MA234.1	CBS 141255	*cspA1, kusA::DR-amdS-DR*	Restored *pyrG* in MA169.4	Alazi *et al.*, 2016
XY1.1	CBS 143276	*cspA1, pyrG^{378}, creA::pyrG*	*ΔcreA* in AB4.1	Yuan *et al.*, 2006
JC1.5	CBS 143271	*cspA1, Δku70,* P*pgaX-amdS* integrated to the *pyrG* locus	P*pgaX-amdS* in MA299.2	Niu *et al.*, 2015
JN29.2	CBS 143272	*cspA1, Δku70,* P*pgaX-amdS* integrated to the *pyrG* locus*, creA::hygB*	*ΔcreA* in JC1.5	Niu *et al.*, 2015
JN123.1	CBS 143275	*cspA1, Δku70,* P*pgaX-amdS* integrated to the *pyrG* locus*, gaaX::hygB*	*ΔgaaX* in JC1.5	Niu *et al.*, 2017
JN130.4		*cspA1, Δku70,* P*pgaX-amdS* integrated to the *pyrG* locus, *gaaR^{W361R}* integrated to the *gaaR* locus	*gaaR^{W361R}* in JC1.5	This study
JN103.1		*cspA1, Δku70,* P*pgaX-amdS* integrated to the *pyrG* locus*, creA::hygB, UV mutagenized yielding to gaaR^{W361R}*	*gaaR$^{W361R-UV}$* in JN29.2	This study
JN129.1		*cspA1, Δku70,* P*pgaX-amdS* integrated to the *pyrG* locus*, creA::hygB, gaaR^{W361R}* integrated to the *gaaR* locus	*gaaR^{W361R}* in JN29.2	This study
SO1.1		*cspA1, pyrG^{378}, kusA::DR-amdS-DR, gaaR::AOpyrG*	*ΔgaaR* in MA169.4	This study
SO2.1		*cspA1, pyrG^{378}, kusA::DR-amdS-DR, gaaR^{W361R}* integrated to the *gaaR* locus	*gaaR^{W361R}* in SO1.1	This study
EA34.1		*cspA1, pyrG^{378}, kusA::DR-amdS-DR, gaaR^{W361R}* integrated to the *gaaR* locus, ectopically integrated pAB4.1	Restored *pyrG* in SO2.1	This study
EA31.1		*cspA1, pyrG^{378}, kusA::DR-amdS-DR, gaaR-eGFP* integrated to the *gaaR* locus	*gaaR-eGFP* in SO1.1	This study
EA36.1		*cspA1, pyrG^{378}, kusA::DR-amdS-DR, gaaR-eGFP* integrated to the *gaaR* locus, ectopically integrated pAB4.1	Restored *pyrG* in EA31.1	This study
EA32.1		*cspA1, pyrG^{378}, kusA::DR-amdS-DR, gaaR^{W361R}-eGFP* integrated to the *gaaR* locus	*gaaR^{W361R}-eGFP* in SO1.1	This study
EA39.1		*cspA1, pyrG^{378}, kusA::DR-amdS-DR, gaaR^{W361R}-eGFP* integrated to the *gaaR* locus, ectopically integrated pAB4.1	Restored *pyrG* in EA32.1	This study
JN35.1	CBS 141256	*cspA1, kusA::DR-amdS-DR, gaaR::hygB*	*ΔgaaR* in MA234.1	Alazi *et al.*, 2016
JN36.1	CBS 143259	*cspA1, gaaR::hygB*	*amdS* loopout in JN35.1	Alazi *et al.*, 2016
EA19.2	CBS 143267	*cspA1, gaaR::hygB, amdS^{+}, ectopically integrated* P*gaaR-eGFP-gaaR*	*eGFP-gaaR* in JN36.1	Alazi *et al.*, 2018
EA29.14		*cspA1, gaaR::hygB, amdS^{+}, ectopically integrated* P*gaaR-gaaR-eGFP*	*gaaR-eGFP* in JN36.1	This study
EA30.6		*cspA1, gaaR::hygB, amdS^{+}, ectopically integrated* P*gaaR-gaaR-(GA)$_4$-eGFP*	*gaaR-(GA)$_4$-eGFP* in JN36.1	This study

Table S2. Primers used in this study. Fusion PCR overlapping regions are written in bold.

Primer name	Sequence (5' to 3')	Used for
gaaX_P5f	CGTTGGTTCGATGTAAATGGG	Amplification and sequencing of *gaaX*
gaaX_GSP10r	TGTATGTGGCATTGATGGAACC	Sequencing of *gaaX*
gaaX_GSP11f	GGGTTGTTTGTCGGTGTACTCG	Sequencing of *gaaX*
gaaX_GSP12r	CGGTTCTTGGTCTTCAGGTCCT	Sequencing of *gaaX*
gaaX_GSP13f	CCTGTACTCTTTGCGCAGTCCT	Sequencing of *gaaX*
gaaX_GSP14r	GCTGTCCCATGATGTGGAGG	Sequencing of *gaaX*
gaaX_GSP15f	TTTCGCCCATCACGCATC	Sequencing of *gaaX*
gaaX_GSP16r	CCATGCATCGACATCTTCAATC	Sequencing of *gaaX*
gaaX_GSP17f	AGTGTACCTGGACTTGACGC	Sequencing of *gaaX*
gaaX_GSP9r	CAAACGTGGAGAAGCCGGT	Amplification and sequencing of *gaaX*
gaaRP7f	TCCTCTTCGGCTTCTGCTTC	Amplification and sequencing of *gaaR*
gaaRP9r	GCATACTCCAGGCTCCCTTG	Sequencing *gaaR*
gaaRP10f	CCTACATGGGAAGCCCTATTGG	Sequencing *gaaR*
gaaRP11r	CGATAGAGCAGACAGTGCACGA	Sequencing *gaaR*
gaaRP12f	CCCTGTTGGTCGACATTACGC	Sequencing *gaaR*
gaaRP13r	AAATCGGGTCGGTCCAGTG	Sequencing *gaaR*
gaaRP14f	AGGACTTCCTGGACCGGTAT	Sequencing *gaaR*
gaaRP8r	CATGTGATCATTTCGTGGCCT	Amplification and sequencing of *gaaR*
gaaRP5f	TGGACGGCGTATGGGATT	Amplification of P*gaaR*-*gaaR*-T*gaaR*
gaaRP6r	CCCAAAGACGGGTGGTAGAGT	Amplification of P*gaaR*-*gaaR*-T*gaaR*
gaaR SBfor	CCTCGACGCCATTCCAGTT	Amplification of Southern blot probe
gaaR SBrev	GGTCATGGACACCGCATTG	Amplification of Southern blot probe
GaaR_GFP4F	**GCATGGACGAGCTGTACAAG**TAAGAAGCCGAATATACGAGCCAT	Amplification of T*gaaR*
GaaR_GFP3R	GCGTGCATCAAGGCGATT	Amplification of T*gaaR*
GaaR_GFP1F	**ATGGTGAGCAAGGGCGAG**	Amplification of *eGFP*

GaaR_GFP1R	**CTTGTACAGCTCGTCCATG**	Amplification of *eGFP*
GaaR_GFP2F	TGGTGTGATGAAGGCAGTGTG	Amplification of P*gaaR-gaaR(-(GA)$_4$)*
GaaR_GFP5R	**TCCTCGCCCTTGCTCACCA**TAGGATTCTCCACCTCCACC	Amplification of P*gaaR-gaaR*
eGFP-gaaR-For	TGGTGTGATGAAGGCAGTGTG	Fusion PCR to amplify P*gaaR-gaaR(-(GA)$_4$)-eGFP*-T*gaaR*
eGFP-gaaR-Rev	TGGGTGTTTGGTGGGTTGA	Fusion PCR to amplify P*gaaR-gaaR(-(GA)$_4$)-eGFP*-T*gaaR*
GaaR_GFP6R	**TCCTCGCCCTTGCTCACCA**TAGCGCCAGCGCCAGCGCCAGCGCCAGGATTCTCCACCTCCACC	Amplification of P*gaaR-gaaR-(GA)$_4$*
gaaRP1f	GCTGCTGCTGCTGCTTTACA	Amplification of *gaaR* 5' flank to create SO1.1
gaaRP2r	**CAATTCCAGCAGCGGCTT**GGCATTGCCTGTGCATAGGA	Amplification of *gaaR* 5' flank to create SO1.1
gaaRP3f	**ACACGGCACAATTATCCATCG**GAAGCCGAATATACGAGCCA	Amplification of *gaaR* 3' flank to create SO1.1
gaaRP4r	GCGTGCATCAAGGCGATT	Amplification of *gaaR* 3' flank to create SO1.1
AOpyrGP12f	**AAGCCGCTGCTGGAATTG**	Amplification of *AOpyrG* 5' flank to create SO1.1
AOpyrGP15r	CCGGTAGCCAAAGATCCCTT	Amplification of *AOpyrG* 5' flank to create SO1.1
AOpyrGP13r	**CGATGGATAATTGTGCCGTGT**	Amplification of *AOpyrG* 3' flank to create SO1.1
AOpyrGP14f	ATTGACCTACAGCGCACGC	Amplification of *AOpyrG* 3' flank to create SO1.1
sgRNAP1	TAATACGACTCACTATAGGGTGGATACGTACTCCTTTTAGTTTTAGAGCTAGAAATAGCAA	Amplification of the DNA template for guide RNA
OTL19	GCACCACCGACTCGGTGC	Amplification of the DNA template for guide RNA
Fw_LIC2	CAACCTCCAATCCAATTTGACTCCGCCGAACGTACTG	Amplification of the DNA template for guide RNA
Rev_P1	**TAAAAGGAGTACGTATCCAC**GACGAGCTTACTCGTTTCG	Amplification of the DNA template for guide RNA
Fw_P1	**GTGGATACGTACTCCTTTTAGTTTTA**GAGCTAGAAATAGCAAG	Amplification of the DNA template for guide RNA
Rev_LIC2	ACTACTCTACCACTATTTGAAAAGCAAAAAAGGAAGGTACAAAAAAGC	Amplification of the DNA template for guide RNA
for_pTE1	CCTTAATTAAACTCCGCCGAACGTACTG	Introduction of *Pac*I site to the DNA template for guide RNA
rev_pTE1	CCTTAATTAAAAAAGCAAAAAAGGAAGGTACAAAAAAGC	Introduction of *Pac*I site to the DNA template for guide RNA
PgaaR_seq_P2	CATCCATCTTAGTCCACCGGC	Amplification of *gaaR*
TgaaR_seq_P2	TCTAGTATTCACTTGTCCCTAA	Amplification of *gaaR*

Table S3. Mutations in *gaaX* in the UV mutants showing constitutive production of pectinases. aa and gDNA represents amino acid and genomic DNA, respectively.

Strain	Type of mutation	Mutation	Codon change	aa change	Predicted length of the mutated protein (aa)	Remarks
S1	Frameshift	Insertion of G	GTT-GGT	V653G	664	gDNA sequenced, no mutation found in *gaaR* (Niu *et al.*, 2017)
S3	Missense	T-C	CTG-CCG	L111P		
S5	Nonsense	C-T	CAA-TAA	Q369Stop	368	gDNA sequenced, no mutation found in *gaaR* (Niu *et al.*, 2017)
S6						No mutations found in *gaaX* or *gaaR*
S7	Missense	T-C	CTG-CCG	L676P		gDNA sequenced, no mutation found in *gaaR* (Niu *et al.*, 2017)
S8	Frameshift	Deletion of TG	GTG-GGG	V193-G	197	
S9	Nonsense	C-T	CGA-TGA	R425Stop	424	
S10	Frameshift	Deletion of G	GGA-GAG	G644E	702	
S11	Frameshift	Insertion G	GGAGCC-GGGAGC	GA469GS	664	
S12	Nonsense	C-T	CAA-TAA	Q369Stop	368	
S13	Nonsense	G-T	GAG-TAG	E129Stop	128	
UV 1	Nonsense	G-T	GAG-TAG	E458Stop	457	gDNA sequenced, no mutation found in *gaaR* (Niu *et al.*, 2017)
UV 3	Nonsense	T-A	TTA-TAA	L101Stop	100	No mutation found in *gaaR* (Niu *et al.*, 2017)
UV 4	Nonsense	C-T	CAA-TAA	Q369Stop	368	No mutation found in *gaaR* (Niu *et al.*, 2017)
UV 5	Nonsense	G-A	TGGGGA-TAGGAA	W270Stop	269	No mutation found in *gaaR* (Niu *et al.*, 2017)
UV 6	Missense	GT-AA	GTA-AAA	V684K		No mutation found in *gaaR* (Niu *et al.*, 2017)
UV 8	Missense	G-A	GGA-GAA	G526E		gDNA sequenced, no mutation found in *gaaR* (Niu *et al.*, 2017)
UV12	Missense	A-G	AAT-GAT	N438D		No mutation found in *gaaR* (Niu *et al.*, 2017)
UV13	Frameshift	Insertion of G	GCG-GGC	A308G	438	No mutation found in *gaaR* (Niu *et al.*, 2017)
UV14	Missense	A-G	AAT-GAT	N521D		
UV15	Missense	GT- AA	GTG-AAG	V193K		
UV16	Missense	GAC-ATT	GAC-ATT	D640I		
UV17	Missense	T-A	GAT-GAA	D496E		
UV18	Nonsense	T-A	TTA-TAA	L548Stop	547	
UV19	Missense	T-A	ATT-AAT	I190N		
UV20	Missense	G-A	GGG-GAC	G194D		
UV21	Nonsense	C-T	CAG-TAG	Q106Stop	105	
UV22	Nonsense	G-A	TGG-TAG	W270Stop	269	
UV23	Nonsense	A-T	AAA-TAA	K435Stop	434	
UV24	Nonsense	G-T	GAG-TAG	E43Stop	42	
UV25	Nonsense	T-A	TTA-TAA	L38Stop	37	
UV26	Missense	A-T	AAT-ATT	N521I		
UV27	Nonsense	A-T	AAG-TAG	K415Stop	414	
UV28	Missense	A-T	AAC-TAC	N378Y		
UV29	Missense	G-C	GCG-CCG	A321P		
UV30	Missense and frameshift	CA-G	TCCACG-TCGCGC	ST426SR	445	
UV31	Missense and nonsense	AC-CT	TTACAA-TTCTAA	LQ101FStop	101	

UV32	Missense	A-C	TAC-TCC	Y638S		
UV33	Missense and nonsense	AC-CT	TTACAA-TTCTAA	LQ101FStop	101	
UV34	Frameshift	Deletion of T	TTT-TTG	F466L	516	
UV35	Missense	A-G	AAT-GAT	N438D		
UV36	Missense	GT-AA	GTC-AAC	V51N		
UV37	Missense and frameshift	CCC-TC	GGCCCG-GGTCGC	GP579GR	593	
UV38	Missense	T-G	CTG-CGG	L607R		
UV39	Missense	G-A	GGG-GAG	G194E		
UV40	Missense	A-G	AAT-GAA	N438E		
UV41	Missense or nonsense	A-T	ATG-TTG	M1L	- (if nonsense)	
UV42	Nonsense	C-A	TCG-TAG	S306Stop	305	
UV43	Nonsense	AC-CT	CAA-TAA	Q102Stop	101	
UV44	Missense	T-A	CTG-CAG	L565Q		
UV45	Nonsense	C-T	CGA-TGA	R349Stop	348	
UV46	Nonsense	C-T	CAG-TAG	Q269Stop	268	
UV47	Nonsense	T-A	TTA-TAA	L101Stop	100	
UV48	Nonsense	A-T	AAA-TAA	K293Stop	292	
UV49	Frameshift	Deletion of C	CCG-CGC	P513R	516	
UV50	Nonsense	C-T	CAG-TAG	Q269Stop	268	
UV51						No mutations found in *gaaX* or *gaaR*
JN103.1						No mutation found in *gaaX*, mutation found in *gaaR*
UV53	Nonsense	AC-TT	CAA-TAA	Q102Stop	101	
UV54	Nonsense	T-G	TAT-TAG	Y535Stop	534	
UV55						No mutations found in *gaaX* or *gaaR*
UV56	Nonsense	C-T	CAG-TAG	Q269Stop	268	
UV57	Nonsense	C-A	TCG-TAG	S593Stop	592	
UV58	Missense	T-C	CTG-CCG	L345P		

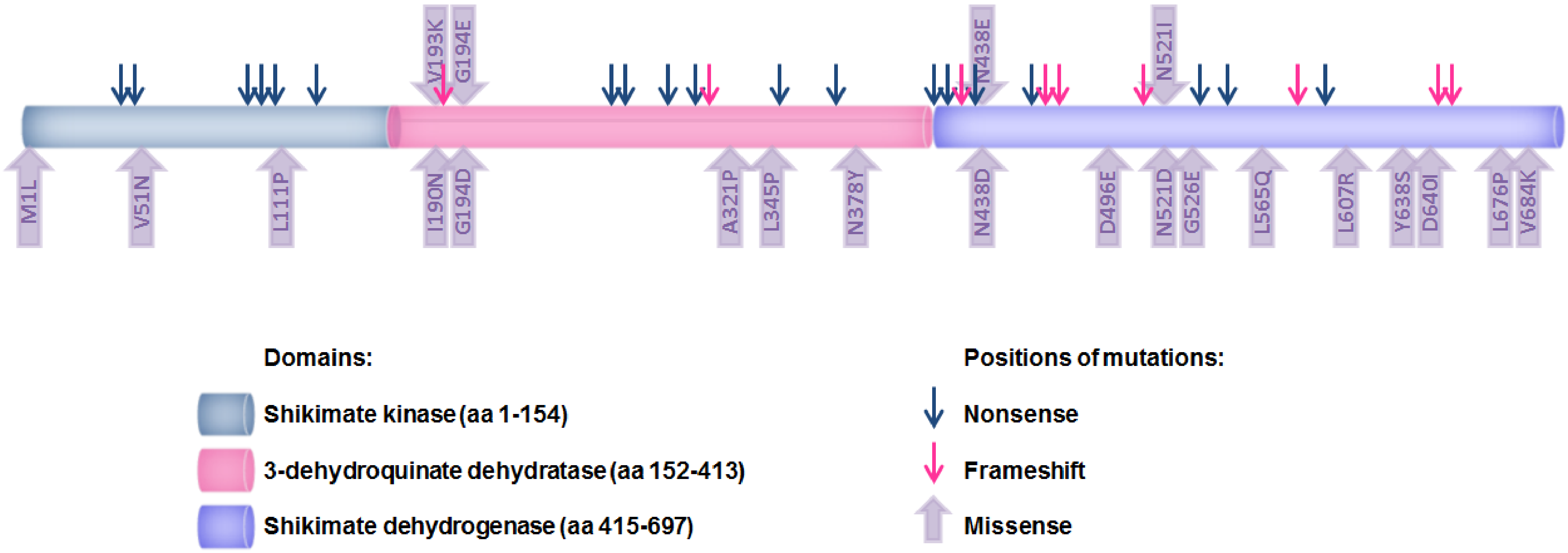

Figure S1. Schematic representation of the domains present in GaaX and mutations in the UV mutants showing constitutive production of pectinases. Domains in GaaX were assigned based on multiple alignment of protein sequences of *A. niger* AROM (NRRL3_11273) C-terminal part (aa 863-1856) and GaaX (NRRL3_08194), and the *E. coli* shikimate kinase (AroL, NP_414922.1), 3-dehydroquinate dehydratase (AroD, NP_416208.1) and shikimate dehydrogenase (AroE, NP_417740.1), using COBALT (Papadopoulos and Agarwala, 2007).

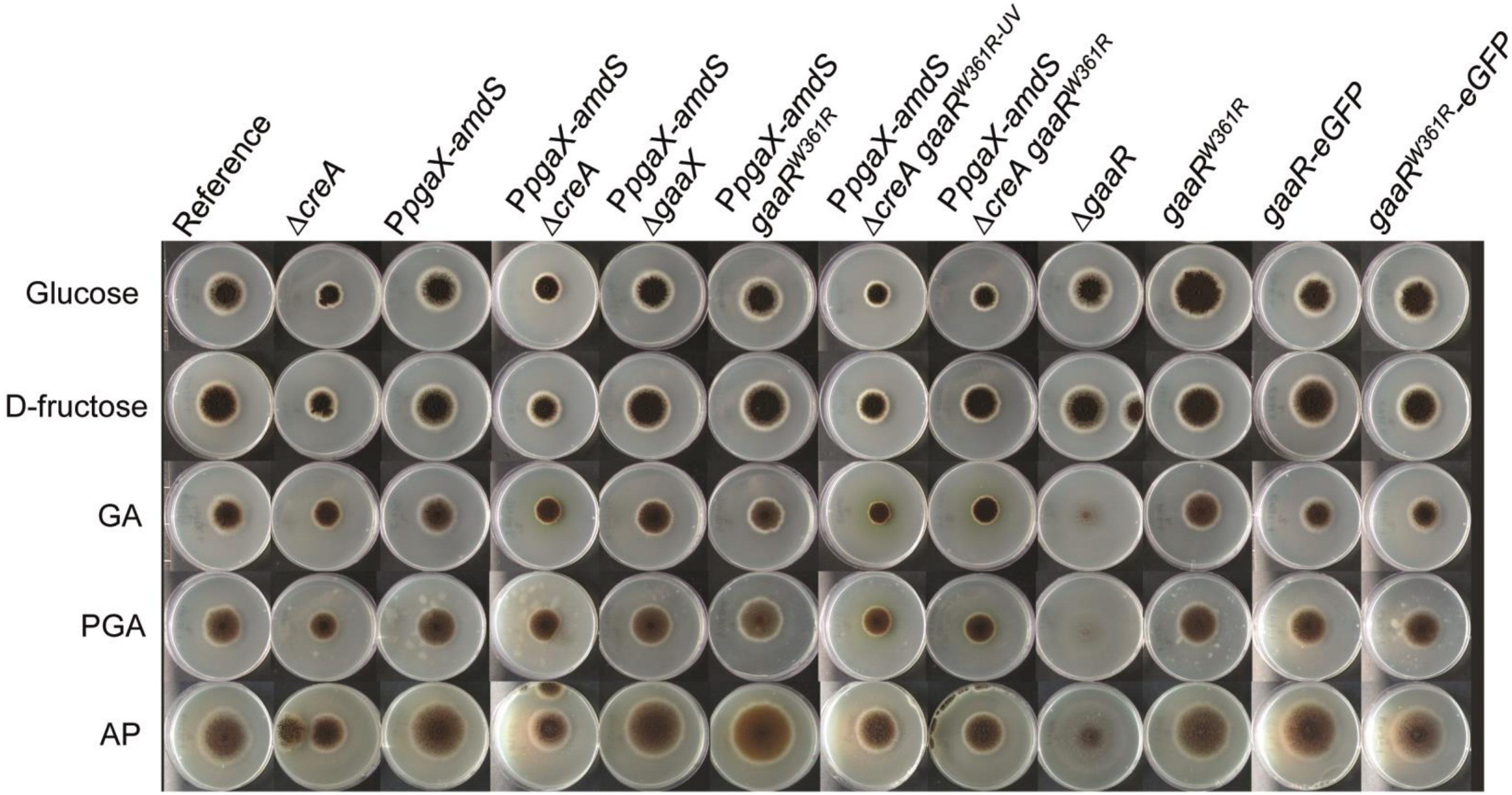

Figure S2. Radial growth assay of the reference (MA234.1), *ΔcreA* (XY1.1), P*pgaX-amdS* (JC1.5), P*pgaX-amdS ΔcreA* (JN29.2), P*pgaX-amdS ΔgaaX* (JN123.1), P*pgaX-amdS gaaR*^{W361R} (JN130.4), P*pgaX-amdS ΔcreA gaaR*$^{W361R\text{-UV}}$ (JN103.1), P*pgaX-amdS ΔcreA gaaR*^{W361R} (JN129.1), *ΔgaaR* (SO1.1), *gaaR*^{W361R} (SO2.1), *gaaR-eGFP* (EA31.1), *gaaR*^{W361R}-*eGFP* (EA32.1), and strains on solid MM containing 50 mM glucose, D-fructose or GA, or 1% PGA or pectin as the carbon source after 7 days at 30 °C.

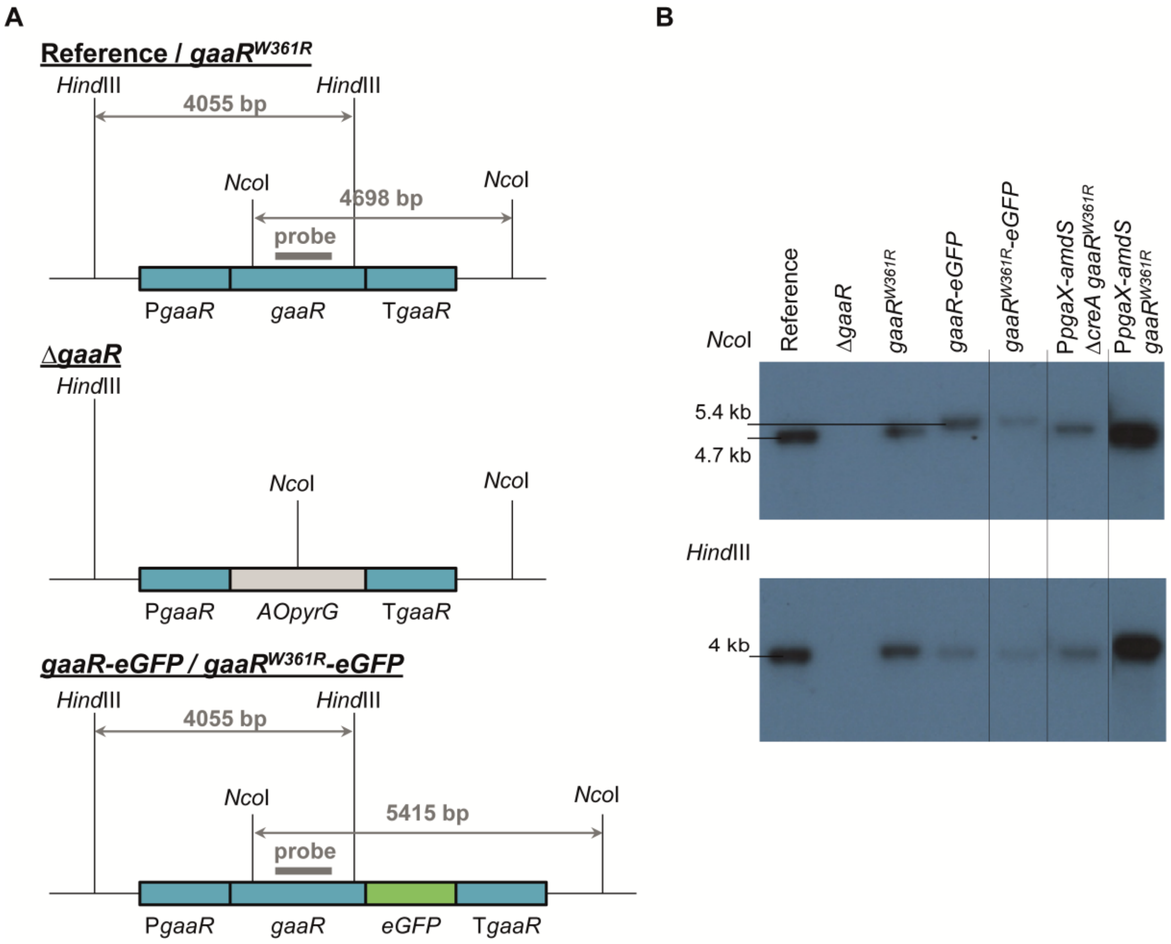

Figure S3. Southern blot analysis of genomic DNA to verify the integration of the *gaaR*^{W361R}, *gaaR-eGFP* and *gaaR*^{W361R}*-eGFP* constructs to the *gaaR* locus in the strains created in this study. **A** Schematic representation of the target gene locus in the reference (MA234.1), *ΔgaaR* (SO1.1), *gaaR*^{W361R} (SO2.1), *gaaR-eGFP* (EA31.1), *gaaR*^{W361R}-*eGFP* (EA32.1), P*pgaX-amdS ΔcreA gaaR*^{W361R} (JN129.1) and P*pgaX-amdS gaaR*^{W361R} (JN130.4) strains. The probe binds to *gaaR* downstream of the *Nco*I restriction site and upstream of the *Hind*III restriction site. Expected band sizes are indicated. **B** Southern blot after hybridization. The lines between genomic DNA samples indicate that the left and right parts of the same blot were combined after removing unnecessary lanes. 4698-bp and 4055-bp bands are visible when the genomic DNA of the reference strain is digested with *Nco*I and *Hind*III, respectively.

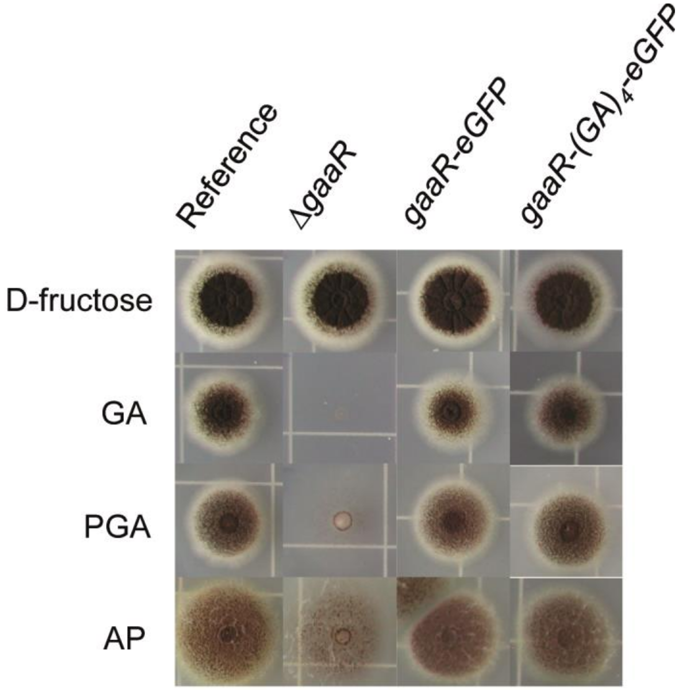

Figure S4. Radial growth assay of the reference (MA234.1), *ΔgaaR* (JN35.1), *gaaR-eGFP* (EA29.14) and *gaaR-(GA)*$_4$*-eGFP* (EA30.6) on solid MM containing 50 mM D-fructose or GA, or 1% PGA or pectin as the carbon source after 5 days at 30 °C.

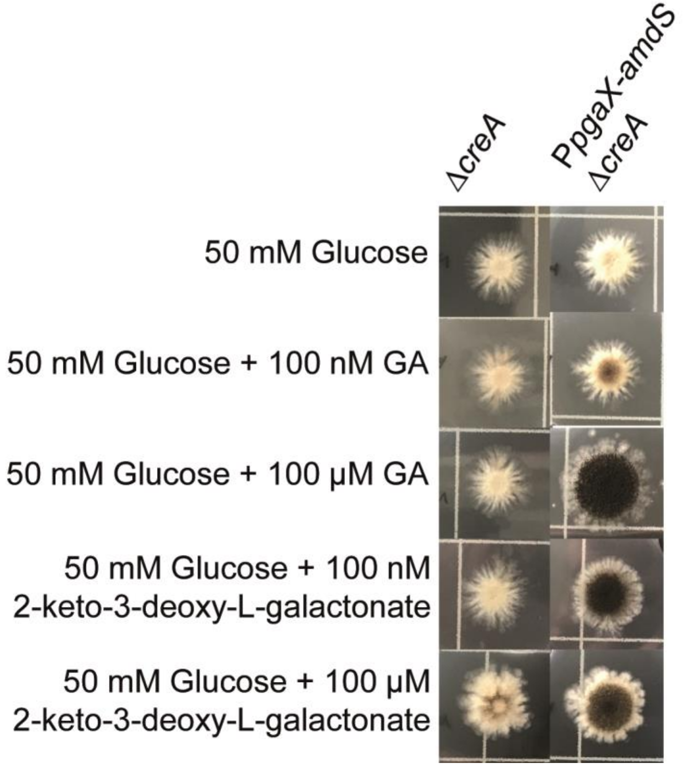

Figure S5. Growth phenotype of the *ΔcreA* (XY1.1) and P*pgaX-amdS ΔcreA* (JN29.2) strains after 10 days at 30 °C on solid MM containing 50 mM glucose or 50 mM glucose with 100 nM or 100 µM GA or 2-keto-3-deoxy-L-galactonate as the carbon source. All plates contain 10 mM acetamide as the sole nitrogen source.

```
AfumGaaR(1-798)       ----IQ--------YSSDPRRKPA-------ATALLIDITRILGITNRYLAA-----GGV    340
AnidGaaR(1-1160)      ----IP--------YSSDRRKTASSGAGSGACSSLLVDISRILGVTHRYLAA-----GGV    390
AnigGaaR(1-740)       ----IH--------YSPDRRKGPG-------GPALLVDITRILGVTHRYLAA-----GGV    353
AoryGaaR(1-757)       ----IQ--------FTSDRRKGPG-------ATALLVDITRILGVTHRYLAA-----GGV    359
TreXyr1(1-940)        SSINIDSSMTDEFGDSPRAARGAHYECRGRSIFGYFLSLMTILGEIVDVHHAKSHPRFGV    697
AnidXlnR(1-875)       -----------------YRAVGPPIECTGHSMFGYFLPLMTILGGIIDLQQAREHPRYGL    633
AoryXlnR(1-971)       -----------------YRQVGPQVECTGHSMFGFFLPLMTILGEIVDLQQAKEHPRFGR    727
AnigXlnR(1-875)       -----------------YRQVGPPVECTGHSMYGYFLPLMTILGGIVDLHHAENHPRFGL    631
AfumXlnR(1-954)       -----------------YRQAGPPVECTGHSMFGYFLPLMTILGEIVDLQQARNHPRFGL    710
                                                       . :: :  ***       *      *

                       GaaR(W361)
AfumGaaR(1-798)       KGDSHFPWHSLSNLSKIRQELDIWATGTQDVFASVEALF---------------------    379
AnidGaaR(1-1160)      KGDSHFPWHALSNLSKIRQELDLWAAGTHDLFASIEALF---------------------    429
AnigGaaR(1-740)       KGDSHFPWHALSNLSKIRQELDLWAAGTQDLFASIETLF---------------------    392
AoryGaaR(1-757)       KGDSHFPWHALSNLSKIRQELDIWAAGTQDLFASIEALF---------------------    398
TreXyr1(1-940)        GFRSARDWDE--QVAEITRHLDMYEESLKRFVAKHLPLSSKDKEQHEMHDSGAVTDMQSP    755
AnidXlnR(1-875)       TFRSGPDLDQ--YIMAITQQLDAYGQSLKDFEARYINSLALAENE---PPENPHIDHLSP    688
AoryXlnR(1-971)       VFRNSADWDH--QVLEITRQLDTYAQSLKEFEARYTSSLALGAGESEAAIEGSHLDHVSP    785
AnigXlnR(1-875)       AFRNSPEWER--QVLDVTRQLDTYGRSLKEFEARYTSNLTLGATDNEPVVEGAHLDHTSP    689
AfumXlnR(1-954)       AFRNSAECEA--QVLEIARQLDVYAQSLKEFETRYTSSLALGAAETEAAMDGSHPNHVSP    768
                         .    .    :  : :.** :  . : . :

AfumGaaR(1-798)       -G--------------HPESSILLLSKLIYHLVHCLIYRPFLPIDLVELRGTGQHQSWQI    424
AnidGaaR(1-1160)      -G--------------HPESTLLLLSKLIYHLVHCLLYRPFLPIDLAELRGTGQHQSWQI    474
AnigGaaR(1-740)       -G--------------HPESTLLLLSKLIYHLVHCLLYRPFLPIDLVELRGTGQHQSWQI    437
AoryGaaR(1-757)       -G--------------HPESTLLLLSKLIYHLVHCLLYRPFLPIDLAELRGTGQHQSWQI    443
TreXyr1(1-940)        LS-VRTNASSRMTESEIQASIVVAYSTHVMHVLHILLADKWDPINLLDDDDLWISSEGFV    814
AnidXlnR(1-875)       SGRSSSTVGSRVNESIVHTKMVVAYGTHIMHVLYVLLAGKWDPINLLEDHDMWISSESFL    748
AoryXlnR(1-971)       SGRSTSTAGSRVNESIVHTKMVVAYGTHIMHVLHVLLAGKWDPINLLEDHDLWISSESFI    845
AnigXlnR(1-875)       SGRSSSTVGSRVSESIVHTRMVVAYGTHIMHVLHILLAGKWDPVNLLEDHDLWISSESFV    749
AfumXlnR(1-954)       SGRSSSTVESRVNESIVHTKMVVAYGTHIMHVLHILLAGKWDPINLLDDNDLWISSESFV    828
                       .                   ::  .. : *::: *:   : *::* :  .    ..  :
                   XlnR (V756)  Xyr1(A824)
AfumGaaR(1-798)       EATNLCFSHSNAIAELVELARNSPLIEWPAFV-GYCVCTAGTVHVHGVHYKGR-EGEVFS    482
AnidGaaR(1-1160)      EATTLCFSHANAIAELVELARHAPRIEWPDLV-AYCLTVAGTVHIHGVHYNGRREGEVFA    533
AnigGaaR(1-740)       EATTLCFSHANAIAELVELARHAPRIEWPALV-AYCVCTAGTVHVHGVHYHGR-EGEVFA    495
AoryGaaR(1-757)       EATTLCFSHANAIAELVELARHAPRIEWPDLI-AYCVCTAGTVHIHGVHYNGR-EGEVFA    501
TreXyr1(1-940)        TATSHAVSAAEAISQILEFD---PGLEFMPFFYGVYL-LQGSFLLLLI--ADKLQAEASP    868
AnidXlnR(1-875)       AAMSHAVGAAEAAADILEYD---PDLSFMPFFFGIYL-LQGSFLLLLA--ADKLQGDANP    802
AoryXlnR(1-971)       AAMSHAVGAADAAADILEYD---PDITFMPFFFGIYL-LQGSFLLLLA--ADKLQGDVSP    899
AnigXlnR(1-875)       SAMSHAVGAAEAAAEILEYD---PDLSFMPFFFGIYL-LQGSFLLLLA--ADKLQGDASP    803
AfumXlnR(1-954)       AAMGHAVGAAEAAAEILEYD---PDLSFMPFFFGIYL-LQGSFLLLLT--ADKLQGDASP    882
                       *   ... ::* ::::*     * : :  :. .  :   *:. :      .: :.:.

AfumGaaR(1-798)       SSAEFLTREMHQLTWLGSYWAGVQHQREMLQTIYTCHAELV-------------------    523
AnidGaaR(1-1160)      SSPDFLNREMSQLDWLGGFWSGVRHHRDLLRTLEVCHAELV-------------------    574
AnigGaaR(1-740)       SSADFLAREMHQLSWLRQYWAGVQHQREMLQSISACHAELV-------------------    536
AoryGaaR(1-757)       SSADFLTREMNQLIWLRHSCSGVQHQREMLQAISACHADLV-------------------    542
TreXyr1(1-940)        --------------------SVIKACETIVRAHEACVVTLSTEYQRNFSKVMRSALALIR    908
AnidXlnR(1-875)       --------------------SVVRACETIVRAHEACVVTLNTEYQRTFRKVMRSALAQVR    842
AoryXlnR(1-971)       --------------------SVVRACETIVRAHEACVVTLNTEYQRTFRKVMRSALAQVR    939
AnigXlnR(1-875)       --------------------SVVRACETIVRAHEACVVTLNTEYQRTFRKVMRSALAQVR    843
AfumXlnR(1-954)       --------------------SVVRACETIVRAHEACVVTLNTEYQRTFRKVMRSALAQVR    922
                                          : ::  . ::::  .* . *
```

Figure S6. Alignment of protein sequences of GaaR from *A. fumigatus* (XP_752182.1), *A. nidulans* (CBF77703.1), *A. niger* (XP_001401443.2), and *A. oryzae* (AO090023000946), and XlnR/Xyr1 of *T. reesei* (EGR48040.1*), *A. nidulans* (CAC81360.1), *A. oryzae* (Q2UD93.2), *A. niger* (CAK42534.1), and *A. fumigatus* (Q4WZV6.1) using Clustal omega (Sievers *et al.*, 2011). Only regions surrounding the amino acids of interest are shown. * The sequence of Xyr1 in the sequence was manually modified by inserting the 20 amino acids sequence (VSLASPSNQFQLQLSQPIFK) in between residues G (319) and Q (320) to correct the gene model.

```
Zymosep_111379_mod_JGI_101685                     ------------------------------------------------------------      0
Aurpu_var_pul1_409643                             ------------------------------------MATRVQV-----------------      7
jgi|CocheC4_1|100143|replaces_COCCC4DRAFT_33348   ---------------------------------------MVQT-----------------      4
Tr_5296                                           ------------------------------------MA-AVQQL----------------      7
FoxG_05376                                        ------------------------------------MA-AVQSI----------------      7
Fgram_10031                                       ------------------------------------MA-AVQSM----------------      7
MGG_12392                                         -----------------------------------MMA-AVQQA----------------      8
NCU_04298                                         ------------------------------------MV-SIQQA----------------      7
Spoth2p4_46981                                    ------------------------------------MA-AVQQL----------------      7
Bcin_P100270                                      MEYIRYQFHLPRLTTFVSKLAQSSTFSLNSDVYHLYCQKYFKPVYPGKTSSSSILTTGKI      60
OIDMA_113116                                      ------------------------------------------------------------      0
TSTA_071040                                       ---------------------------------------------------MALV-----      4
Pc22g17290                                        -----------------------------------MIPTAF-PQ----HMATPLL-----      15
Afu_4906460                                       -----------------------------------MMQTAIH-Q----PMAMTIL-----      15
AN10544                                           -----------------------------------MMQTAVHHQ----PVATTLR-----      16
An04g00790                                        -----------------------------------MMQTALH-Q----PMGMTLL-----      15
AOR_1_1674144                                     -----------------------------------MMQTAIH-K----PVAMTLL-----      15

Zymosep_111379_mod_JGI_101685                     -------------------------------MAVGIE-------------SLTALKALRV      16
Aurpu_var_pul1_409643                             -------------------------E-T---------------STMSFTKPRPIVAVEEV      26
jgi|CocheC4_1|100143|replaces_COCCC4DRAFT_33348   -------------------------AGG---------------MTSPHLKEGMVIDVQEI      24
Tr_5296                                           ---LASPSP-----TP---APISSQHINNGSAAANMEEPGTADHDPV--VIDSGLMKGAP      54
FoxG_05376                                        ---LAETLP-----GP---TPSIALNGATEPTTMHIDR---PSSSPV--MVNESTFGSTP      51
Fgram_10031                                       ---IAESLP-----AP---TPTIALNGSTDPAAMQIDE---PSSSPA--TISDSTFGCTP      51
MGG_12392                                         ---FVVTAP-----AAPAPVRPKSLNGTANDPRMDLDD---KPHQFT--LENGATFGSGL      55
NCU_04298                                         ---EPAPAL-----IS----APVPSTIP-AHRAIDLVK---GRTTSP--AVGNLPLLRGP      49
Spoth2p4_46981                                    ---EPVPTP-----AA----RPQSIADS--PFTNNGDT---ERASPP--AQPGISLCSEP      48
Bcin_P100270                                      IRKEAFVRNSQVHQELPQKFSNLSIINSSESMATKQQQSSPTPTRPKSTN--GIINGSKA      118
OIDMA_113116                                      ------------------------------MATKQQQQSAPTPARPKSTN--GVINGTKA      28
TSTA_071040                                       --SS--------------------------I-TITED-LRTPTSGITLT--SSHEPTPAA      32
Pc22g17290                                        --DDPPVSTALKAQDPPGTN--PNIAGPSNA-TTTLS-LAPPASGPTDQT--AAGIAAPA      67
Afu_4906460                                       --ADTAPSVTN-----------NSASAPSPA-NGA----HPPLATAVSANSAVAAAPPSV      57
AN10544                                           --ADSPVSVAS-----------TSLKGTS--------------------ASAALPFPQAL      43
An04g00790                                        --ADAAT-SSL-----------TALKGST--------------------T-SSALTSLTV      40
AOR_1_1674144                                     --TETSS-P-V-----------TSIKGPS--------------------SFAITSPVQNL      40

                                                          V51N
Zymosep_111379_mod_JGI_101685                     SGHQLPRLLVMFDHGQEWIARLIADVLGYTCRFVDEESAMGSLGEVAVIGVEGCNVR---      73
Aurpu_var_pul1_409643                             RGHEEPSILVLYERNQEQIVAEIAGVLGQQWRLVSPGDNEPCPVEGTVIGVSDAHDL---      83
jgi|CocheC4_1|100143|replaces_COCCC4DRAFT_33348   PHGARPALLILHNEGEESIVDTIADVLGQRWELASSMEDALAGPKETVYGLSSLVAK---      81
Tr_5296                                           QPGDSRIVVIVYGQGQEHVVSVFAEVLGKPVKIKRHFRDVDHDDEGFVIGLALDEAQ---      111
FoxG_05376                                        AKGDSRIVVIVYGPGQEHIVSVFAEVLGTPCRTRNGFEEVSSEDSGFVVGVAANEAK---      108
Fgram_10031                                       TRGDSRIVVIVFGPGQDKIVSVFAEVLGTPCRIRNGFDKVSADDSGFVVGVVAGEAK---      108
MGG_12392                                         QRGDTRIVTIIYSPGQERILSVFAEVLGQPCRLGASFSNVTLADQGAVVGILASDAA---      112
NCU_04298                                         RNGDTRTAVIVFGQGQGNIVAVFAEVLGKSYRVRNGFRDVSSDDRDLVVGIAATQAK---      106
Spoth2p4_46981                                    QRGDTRIVVIMFGKGQEKIVSVFAEVLGKPSKMKPRFESITKDDQGWVIGIPADSAK---      105
Bcin_P100270                                      GMGDERIVVIVFGQGQDRIVPVFADVLGKPHRMATSFSAVGDEDHGTVIGIAASDAK---      175
OIDMA_113116                                      GEGDSRIVVIIYGQGQEKIVSVFASVLGKPYRLASGFSAVGAEDHGTVIGVAAADAE---      85
TSTA_071040                                       SPPIKRTVVIVYDIGQENILQVVAEVLGKAWTTVTTLLRVVLGSDAVVVGILNRDLEKTA      92
Pc22g17290                                        AATAAGTVVIIYGQDQEHIVRLVAEVLGKPWTTDASLGMLARANYAAVVGILAHDLA---      124
Afu_4906460                                       PADPPRTVVVVYRSGQDSIVHLVAEVLGKPWTTETSLSTLVTGSNAVVVGILAAELA---      114
AN10544                                           PQEPSRTVVAVYGPGQDAIVQVVADILGKPWTTETSLSTLGRGSSAVVVGIRATDLP---      100
An04g00790                                        PPEPARTVVTVYTPGQEAIVQVVADILGKPWTTETSLSTFGRGSNAVVVGFLATEDSI--      98
AOR_1_1674144                                     PPEPSRAVAIVYGPNQDTIVQVVAEILGKPWTTESSLSTLGRGSNAVVTGILADDLG---      97
                                                            :.  .:  :   .* :**                   * *.

                                                           L111P
Zymosep_111379_mod_JGI_101685                     -DVKGL---DRVVLATHCIDEGE-RDGKLIQACDYEFLYSQNDPATSRKELVRFLGFTLG      128
Aurpu_var_pul1_409643                             SQVQRWR-GSSTILATHCLDDRNPAVGSRSSICDYEYLYT-TKPF-LRRDLARFLSFILG      140
jgi|CocheC4_1|100143|replaces_COCCC4DRAFT_33348   PHLDSWD-RSRLLINTHRVDGSYELDESITDRCDYEYLYT-RSPF-FRRDVSRYLSFILG      138
Tr_5296                                           RDVEDRDQQLLVAINAHC------------------------------------------      129
FoxG_05376                                        GDVAARNRNLVVAINTHCVNLGMPPDTQLSTHCDYEFLYT-ESPF-FRRDLARFISFTLG      166
Fgram_10031                                       GDVAARNQGLVVAINTHCVNLGMPPDIHLSANCDYEFLYT-ESPY-LRRDLARFISFTLG      166
MGG_12392                                         VDLAGRDRNLVVAINAHCVNLGMPPDPFLSSQCDYEFLYT-NTPF-FRRDLARFISFTLG      170
NCU_04298                                         SDIAARDRTSIVAINVHCTSMGMPPDAYLSAQCDYEFLYT-EALC-SRRDLSRFVSHTLG      164
Spoth2p4_46981                                    DDIATRDRGLVVAINAHCTGLGMPPDVYLSAQCDYEFLYT-ESPF-FRRDLARFTSHILG      163
Bcin_P100270                                      GDIESRNRSLIVAINAHCVALGMPPDLNLSASTDYEFLYT-ETPF-FRRDLSRFIAFILG      233
OIDMA_113116                                      VDIATRNKALVVAINSHCVNLGMPPDVRLSAASDYEFLYT-EQPF-FRRDLARFIAFTLG      143
TSTA_071040                                       PEMRVDDFSRLLIVNTHCVDDGSFPDARLSEGCHYEFIYS-QNLF-FRRDLTRFLSLILG      150
Pc22g17290                                        RSLEGCERSSLVLVNTYCVXNGFTPDDSLTESCDYEFLYS-RSPF-LRRDLSRFLSLIFG      182
Afu_4906460                                       RSLDECDPSTLIVINTHCVDDGSSPDEALTDRCDYEFLYS-HSPF-LRRDLTRFLSLILG      172
AN10544                                           GSLDGCDKSACMLVNLHCVDDGSFPEEGLTELCDYEFLYSLRSPF-FRRDLTRFLSLILG      159
An04g00790                                        PSLQVDDQTACVLVNTHCVDDGSFPDDALTERCHYEFLYSLRSPF-FRRDLTRFLSLILG      157
AOR_1_1674144                                     RALEGCEKPAGILINTHCVDDGSFPDESLTDRCDYEFLYSLRSPF-FRRDLTRFLSLILG      156
                                                    :          :  :
```

Figure S7. Alignment of protein sequences homologous to GaaX present in 18 Pezizomycetes species using Clustal omega (Sievers *et al.*, 2011). Missense mutations in *A. niger* GaaX and conservation in other species are highlighted.

```
                                                                                  I190N V193K G194D/E
Zymosep_111379_mod_JGI_101685                     QAKPHDEISRKQRSTMLSLALPDVRRALPDLDIVSIGADAIELRVDLLRDTTG---DGQY   185
Aurpu_var_pull_409643                             QINPHKDLAKKAKTTLISTTFPDVHAALPNLDILSVGADAIEIRVDLLKETKS---DGSV   197
jgi|CocheC4_1|100143|replaces_COCCC4DRAFT_33348   QIGPHRDLTKKARTTLISTTFPDVHAALPNLDILSVGADAIEIRVDLLQEPLA---DGAI   195
Tr_5296                                           -------LMAKPRTFFISTTFPDVRAALPNLDILTVGADAVEIRVDLLREPRA---DGTF   179
FoxG_05376                                        QINYHEALMAKPRTYFISTTFPDVHAALPNIDILTVGADAVEIRVDLLREPLG---DGSY   223
Fgram_10031                                       QINCHEALMAKPRTYFISTTFPDVRAALPNIDILTVGADAVEIRVDLLREPLS---EGGF   223
MGG_12392                                         QINHHETLMTKPRTFMISTTFPDVRAALPNIDILTVGADAVELRVDLLEEPLA---EGGH   227
NCU_04298                                         QLNHHETLMAKPRTYFISTTFPDVHAALPNIDILTVGADAVEIRVDLLKEPLA---DGTY   221
Spoth2p4_46981                                    QICHHRALMAKPRTFIISTTFPDVHAALPNIDILTVGADAIEIRVDLLKEPLG---DGTY   220
Bcin_P100270                                      QISHHEALITKPRTYFISTTFPDVRTALSNLDILSVGSDAVEIRVDLLKEGLT---DGTF   290
OIDMA_113116                                      QISHHEALITKPRTYFISTTFPDVRTALSNLDILSVGSDAVEIRVDLLKEPLP---DGTF   200
TSTA_071040                                       QRRPHEDLAVKRRTNLISTTFPDVRAALSNLDILSVGSDAIELRVDLLEEPLS---DGSR   207
Pc22g17290                                        QTRPHDDLRTKTRTNFISTTFPDVHAALPNLDILSVGSDAVEIRVDLLVEPVPKG--WTT   240
Afu_4906460                                       QTRPHEDLKTKTRTNFISTTFPDVHAALPNLDILSVGSDAVEIRVDLLVEPSPQ---GVV   229
AN10544                                           QTRPHEDLKTKNRTNFISTTFPDVHAALPNLDILSVGSDAVEIRVDLLVDPDPLPQDQGY   219
An04g00790                                        QTRPHEDLKTKNRTNFISTTFPDVHAALPNLDILSVGSDAVEIRVDLLVDPEPPQ--GAE   215
AOR_1_1674144                                     QTRPHEDLKTKNRTNFISTTFPDVHAALPNLDILSVGSDAVEIRVDLLVEPSP-V--GIS   213
                                                         :  * :: ::* ::***:  ** ::**:::*:**:*:***** :

Zymosep_111379_mod_JGI_101685                     DFIPSLEFVGEQVMLLRQRTDLPLVFTIRPTNGNGRFPTDNPELCYSYLRKAIQWGCEYI   245
Aurpu_var_pull_409643                             SDIPSLQYVGEQLMTLRQRTELPIIFTTRCTKENGRFPMDDPNLFYDYLYRAVQWGCEYI   257
jgi|CocheC4_1|100143|replaces_COCCC4DRAFT_33348   SKVPSLKYVGEQVMVLRQRTELPIIFTTRCTKENGRFPMDDPSLYYRYLARAIQWGCEYI   255
Tr_5296                                           SDIPSLSYVGQQVMLLRQRTELPIIFTTRCTQENGRFPMDKPDLYYEYLYRAIQWGVEYI   239
FoxG_05376                                        SEVPSLSYVGEQVMLLRQRTELPIIFTTRCVRENGRFPMDKPELYYEYLYRAIQWGVEYI   283
Fgram_10031                                       SEVPSLSYVGEQLMLLRQRTELPIIFTTRCVRENGRFPMDRPELYYEYLYRAIQWGVEYI   283
MGG_12392                                         AAVPSLSYVGEQVMLLRQRTELPIIFTTRCTRENGRFPMDNPDLYYEYLYRAIQWGVEYI   287
NCU_04298                                         APVPSLSYVGEQVMLLRQRTELPIIFTTRCTAENGRFPMDNPDLYYEYLYRAIQWGVEYI   281
Spoth2p4_46981                                    SEISSLSYVGEQLMLLRQRTELPIIFTTRCTNENGRFPMDNPGLYYEYLYRALQWGVEYL   280
Bcin_P100270                                      NSVPSLSYVGEQVMLLRQRTELPIIFTTRCTRENGRFPMDNPELYYEYLYRAIQWGCEYI   350
OIDMA_113116                                      SSVPSLSYVGEQVMLLRQRTELPIIFTTRCTRENGRFPMDNPELYYEYLYRAIQWGCEYI   260
TSTA_071040                                       SSIPSLNYVGQQVMLLRQHTELPIIFTTRCVRENGKFPMEDPELFHRYLRRAIQWGIEYI   267
Pc22g17290                                        PPVPSLKYVGQQLMLLRQHTELPIIFTVRCTRENGKFPMDDPALFYKYLRRAIQWGVEYV   300
Afu_4906460                                       SPVPSLRYVGQQVMLLRQHTELPIIFTTRCTKENGKFPMDDPMLFYRYLRRAIQWGCEYI   289
AN10544                                           PGIPSLRYVGQQLMLLRQHTELPIIFTTRCTKENGKFPMDNPALFYRYLRRAIQWGVEYI   279
An04g00790                                        TSVPSLRYVGQQLMLLRQHTELPIIFTTRCTKENGKFPMDDPMLFYKYLRRAIQWGVEYI   275
AOR_1_1674144                                     NRVPSLRYVGQQLMLLRQHTELPIIFTTRCTKENGKFPMEDPGLFYRYLRRAIQWGVEYI   273
                                                    : ** :**:*:* ***:*:**::** * .   **:** : * * : ** :*:*** **:

                                                                                            A321P
Zymosep_111379_mod_JGI_101685                     EIEGSMPEVIRRKLAAEKGNTKIISSWYDLSGAFMWSSPVAERLFRLGAVYGDVVKMVAG   305
Aurpu_var_pull_409643                             DVELWLPEDIRRKLASQKGNSKIISAFHDFSGNFKWTSPDAQRLFEQGAVYGDVVKMIAL   317
jgi|CocheC4_1|100143|replaces_COCCC4DRAFT_33348   DVELWLPEEIRKKLAENKACTKIISAFHDFSGTFKWTSPETQRLFEMGAVYGDIVKMYAL   315
Tr_5296                                           DVELWLPERIRKKLYEKRGNSRIMSAFHDFSGTFKWPSQYAEDVFARSKQYADIVKMIAI   299
FoxG_05376                                        DVELWLPEEIRKKLHEQRGNSRIMSAFHDFSGNFRWPSAYAQEVFQRSIPYADIIKMIAI   343
Fgram_10031                                       DVELWLPEEIRKKLYDQRGNSRIMSAFHDFSGSFRWPLAYAQEVFERSIPYADIIKMIAI   343
MGG_12392                                         DVEVWLPERIRQRLWEKRGSSRIMSAFHDFSGTFKWPSSHAQDIFSESRRLADIVKMHAI   347
NCU_04298                                         DVEIWLPERIRRRLYEQRGSSRIMSAFHDFSGTFKWPSARAEATFRESARFADIVKMIAI   341
Spoth2p4_46981                                    DVELWLPESIRKKLYEQRGNSRIMSAFHDFSGTFKWTSERAESIFLESQRYADCVKMIAI   340
Bcin_P100270                                      DVEVWLPEEIRKKLFEQRGNSRIISAFHDFSGTFKWPSLQAQNIFQESRKYGDIVKMITI   410
OIDMA_113116                                      DVELWLPEDIRRRLFEQRGSSRIISAFHDFSGTFKWPSPQAQSIFEESRKYGDIVKMITI   320
TSTA_071040                                       DVELWLPEEIRKDLSDHRGNSVLMSAFHDFSGKWKWTSPEAQQVFKEGARYADIVKMIAM   327
Pc22g17290                                        DVELWLPEEIRRQLFEKKGHSMILSAFHDFSGTWKWTSEEAQRIFTESAKYADIVKMIAM   360
Afu_4906460                                       DVELWLPEEIRRRLAEQKGHSVIMSAFHDFSGTWKWTSPEAARIFAESARYADIVKMIAM   349
AN10544                                           DVELWLPEDIRRKLAEVKGNSVIMSAFHDFSGEWKWTSPEAPRIFEESAKYADIVKMIAM   339
An04g00790                                        DVELWLPEDIRRRLSEVKGNSIIMSAFHDFSGAWKWTSPEAPRIFAESAKYADIVKMIAM   335
AOR_1_1674144                                     DVELWLPEDIRRQLAEVKGNSIIMSAFHDFSGTWKWTSPEAPRVFAESAKYADIVKMIAM   333
                                                  ::*  :** **: *   :. : ::*:::*:** : *    :   *  .   .* :** :

                                                           L345P                            N378Y
Zymosep_111379_mod_JGI_101685                     TKTHDENYELESFRSMVQRD---YPQALFCGVNVGAVGQPSRTINKVLTPITHPLLKGNA   362
Aurpu_var_pull_409643                             VSTMQENYELEYFRSIIQST---YPYPPFSGLNMGPMGQLSRALNKIFTPITHPLLPIIA   374
jgi|CocheC4_1|100143|replaces_COCCC4DRAFT_33348   VNSMQENYELEYFRSTIQAK---YPHPPFSGLNMGPMGQLSRTLNKIFTPITHPLLPMIA   372
Tr_5296                                           INDHNENFELEYFRSKIRAQ--YPDGPPLSAVNMGEIGQFSRTLNTVFSPITHPLLPIIA   357
FoxG_05376                                        INEHNENFELEYFRSKMRAE--YPNAPPFSAVNMGEVGQFSRTLNPVFTPITHPLLPLIA   401
Fgram_10031                                       INEHNENFELEYFRSKMRAE--YPNAPPFSAVNMGEVGQFSRTLNPVFTPITHPLLPLIA   401
MGG_12392                                         INDHNENFELEYFRVKVKSE--QPGAPPLSAVNMGEVGQFSRTLNRVFTPITHPLLPIIA   405
NCU_04298                                         INDHNENFELEYFRSKIKAE--YPDAPPLSAVNMGETGQFSRTLNRVFTPITHPLLPIIA   399
Spoth2p4_46981                                    INDHNENFELEYFRSKIKAK--YPDSVPLSAVNMGETGQFSRTLNKVFTPITHPLLPIIA   398
Bcin_P100270                                      INSMSENYELEYFRSQIKAN--YPDGPPLSAVNMGQLGQLSRALNTVFSPITHPLLPIVA   468
OIDMA_113116                                      INSMSENYELEYFRSQIKLN--NPDGPPLSAVNMGQLGQLSRALNTFFSPITHPLLPIVA   378
TSTA_071040                                       VNTIEENYELEYFRSTIKA---NYSYPLLSAVNMGPQGQLSRALNTVFSPITHPLLPIIA   384
Pc22g17290                                        VHTIEENYELEYFRSTIKTRGAG---PPLSAVNMGQMGQLSRALNTVFSPITHPLLPMIA   417
Afu_4906460                                       VHTIEANYELEYFRSTVKARGAGDSSPLLSAVNMGQMGQLSRALNTVFSPITHPLLPMIA   409
AN10544                                           VDSVEANYELEYFRSTIKQ--THGTYPLLSAVNMGQLGQLSRALNTVFSPITHPLLPMIA   397
An04g00790                                        VTTIEANYELEYFRSTIKG--TYGPYPLLSAVNMGQLGQLSRALNTVFSPITHPLLPMIA   393
AOR_1_1674144                                     VNTVEANYELEYFRSTIKR--AHGSYPLLSAVNMGQMGQLSRALNTVFSPITHPLLPMIA   391
                                                      . *:***  **  ::          :..:*:*   ** **::*  .::*******    *
```

Figure S7. Alignment of protein sequences homologous to GaaX present in 18 Pezizomycetes species using Clustal omega (Sievers *et al.*, 2011). Missense mutations in *A. niger* GaaX and conservation in other species are highlighted. (Continued)

```
                                                                                    N438E
Zymosep_111379_mod_JGI_101685                     APGEMNAAQINSTLCATSHLSALNIYASSY----KHHHTANFYTQCLNELSLPYTLHT--   416
Aurpu_var_pull_409643                             APGQLSAAEINSALHSMGQMPKLDMYCFGSM---RATGQAMFFEKCINELSLPHQLVCVE   431
jgi|CocheC4_1|100143|replaces_COCCC4DRAFT_33348   APGQLSAAEINAALHTMGQMPKRDIYGIGSF---RSTAQAMFFEKCFNELSLPHSFSFAE   429
Tr_5296                                           APGQMSTAEINAALALLGQLPKKYFYGISSAAARTIAPRAPFYEKCFNELGLPHQFAVVE   417
FoxG_05376                                        APGQLSAAEINAALSLLGQLPRKRIYGINSSSSRNATPQAPFYEKCINELGLPHQFAVVE   461
Fgram_10031                                       APGQLSAAEINAALSLLGQLPRKRIYGITSALSRSATPQAPFYEKCINELGLPHQFAVVE   461
MGG_12392                                         APGQMSAAQINAALAMVGQLPKKHFYGISGPSSRGATPQAPFYEKCFNELGLPHHFSVVQ   465
NCU_04298                                         APGQLSASEINQALSLLGQLPRKNIYGINTPSSRISTPQAPFYGKCFNELGLPHHFSVVE   459
Spoth2p4_46981                                    APGQMSAAEINQALALLGQLPKKNMYGITSPAMRSVTPQAPFYEKCFNELGLPHQFAVVE   458
Bcin_P100270                                      APGQMSAAEINGALATMGQLPKKNIYAIGTF---RTTPQATFFEKCFNELGLPHNFASLD   525
OIDMA_113116                                      APGQMSAAEINGALSTMGQLPKKNIYAVGSF---H-------------------------   410
TSTA_071040                                       APGQLTAAEINRALHIMGQLPKRDMYAIGSF---RSTSQSMFMEKCFNELGLPHNFSCID   441
Pc22g17290                                        APGQLTAAEINEALHIMGQLPKRELYAIGSF---RSTPHSMFMEKCLNELGLPHNFTSVD   474
Afu_4906460                                       APGQLTAAEINEAMHIMGALPKRDLYVIGSF---RATPQSMFMEKCFNELSLPHTLTSID   466
AN10544                                           APGQLTAAEINEALHIMGQLPKRDLYAIGSF---RATPQSMFMEKCFNELSLPHTLTSMD   454
An04g00790                                        APGQLTAAEINEALHIMGQLPKRDLYAIGSF---RSTPQSMFMEKCFNELSLPHTLTSID   450
AOR_1_1674144                                     APGQLTAAEINEALHIMGQLPKRDLYAIGSF---RSTPQSMFMEKCFNELSLPHTLTSID   448
                                                  ***::.:::** ::   . :    :*

Zymosep_111379_mod_JGI_101685                     ---TSPANLSASLGRPNFGGCTITPSLSVKQ------------TPCLPTLSAAASSIGKV   461
Aurpu_var_pull_409643                             QSTQD--GLATVVKVPHFGGAFINPPIAASA-------------PCLPSISDAAASIGQI   476
jgi|CocheC4_1|100143|replaces_COCCC4DRAFT_33348   RGPKA--SIEHILRRNNFGGAYINPPLGASA-------------PYLPSLSNAARAIGQV   474
Tr_5296                                           RQHNNPAGIQAWCNQKDFGGAYLDPGLSYQTIMKHNPWFSTLRNGQGPTLSEAAQGIGII   477
FoxG_05376                                        RHPNNPRSLETWCNQKHFGGAYLNPGLPHNALTKISPFFAGLNGGNGPILTEAARVIGVV   521
Fgram_10031                                       RHPNNPRSIEAWCNQKHFGGAYLNPALPQHTLTKASPFFAGLNGGNGPVLTEAARVIGVV   521
MGG_12392                                         RQARGIGGVDTWCNQKDFGGAYLNPAMSHHDLISKSHFFQSLNSGSGPILTEAALLIGVV   525
NCU_04298                                         RPLKGFASVEAWCNQKNFGGAYLYPAVSFQHLISNSAFFASINNGTGPILSDSVKLIGMV   519
Spoth2p4_46981                                    WQPKGPGCIGTWCNQKNFGGAYFNPAVSLKSLATHSDFLASLNNGAGPTVSEAARLIGMV   518
Bcin_P100270                                      RGVKG--SVEAFCLQPNFGGAYINPPLHSSQ-------------PYIPMLSDAARTIGAV   570
OIDMA_113116                                      ------------------------------------------------------------   410
TSTA_071040                                       RGPRG--SIESVIMQPNFGGAYVNPPLSSSA-------------SYIPTKSDAARAIGLI   486
Pc22g17290                                        RGPKG--SMESVLVQPHFGGAYLNPPVPTTT-------------SFIPAASDAARAIGLV   519
Afu_4906460                                       RGPKG--TIEGVIMQPSFGGASVNPPISAAT-------------PYIPAVSEAARAIGLV   511
AN10544                                           RGPAG--SLERVFSQPSFGGAALHPPLSS-T-------------SHLPAISDAARAIGLV   498
An04g00790                                        RGPSG--SIERVITQPSFGGASVHPPITSGT-------------SSIPAISEAAQAIGLV   495
AOR_1_1674144                                     RGPMG--SIERVITQPSFGGASVHPPISSST-------------TCIPAVSDAARAIGLV   493

                                                D496E                                        N521D G526E
Zymosep_111379_mod_JGI_101685                     DAITVLSTSG-----------------------------------AEPLLADNLTYRAIL   486
Aurpu_var_pull_409643                             DTVVASSDGT-----------------------------------KSSLVGDNATWKGIR   501
jgi|CocheC4_1|100143|replaces_COCCC4DRAFT_33348   DTVLVPPGSS-----------------------------------NASLIGDNTRWKGIR   499
Tr_5296                                           DTIVVKSASSSSASGSGGSTSTPPSQYAS---GTLPPGGQPGLAPNTSLIFDNASWRGIL   534
FoxG_05376                                        DTIVVQSGTSSRSSSP-GSMPSSPRQRQS---DSVDLGSQSGLGSSTSLVFDNAGWKGIL   577
Fgram_10031                                       DTIVVQSGTSSRSSSP-GSIPSSPRHRQS---DSVEFGSQSGLGSTTSLVFDNAGWKGIL   577
MGG_12392                                         DTIVVRPGPASPASIPSTPI-QRSSPALNGNGQGLYQPPASGLPAGTALVFDNALWRGVL   584
NCU_04298                                         DTIVVQSKGAFSASPAPSSAPSSLGRHQRSESIGSVALSQSGLPPTTVLVFENAAWKGIL   579
Spoth2p4_46981                                    DTIVVRPAPSSAPTSAPSSIPSSPPRHKNGDSYSALGPSQSGLPPNTTLVLDNASWKGIL   578
Bcin_P100270                                      DTIVVRGE-----------------------------------GPSSTFIGDNATWKGIR   595
OIDMA_113116                                      ------------------------------------------------------------   410
TSTA_071040                                       DTIAVNGSDG-----------------------------------QHKFIGENATWKGIR   511
Pc22g17290                                        DTIAMTPGEA--------------------------------APGTSRFIGENAAWKGIR   547
Afu_4906460                                       DTIVVGESQSQSQSQSQSQ------------SQSQSQSQPHGPPTARTLIGENATWKGIR   559
AN10544                                           DTIVVTS--------------------------------------PGSLVGENATWKGIR   520
An04g00790                                        DTIVAMRGSGRPQ---------------------------QAPSPQQQLMGENATWKGIR   528
AOR_1_1674144                                     DTIVAANS----------------------------------AAPNAQLVGENATWKGIR   519

                                                                                     L565Q
Zymosep_111379_mod_JGI_101685                     STLTSAFVPSAYTSRSAIVLANNESSAAEALYALGILGVATIYTIGFEATSGIENVM--T   544
Aurpu_var_pull_409643                             ATLCRDFVPSAYSGQAALLLASQESQAAAAIFALRSLNIGPIYTIGFKVQGPLSG---FV   558
jgi|CocheC4_1|100143|replaces_COCCC4DRAFT_33348   ATLTRDFVPSAYSGQAALLLANNEGDASASIFALKNLGIGPIYTIGFKSHGPLST---DV   556
Tr_5296                                           STLTRDLAPSAYFGRTAVVLALTADDAAPVFFALKALKIAKIYTVGFRTPSGLARNAPPI   594
FoxG_05376                                        STLTRDLAPSAYFGRSAVVMASSSDDAAPVFFAMRALKISKIYTVGFRTPSNLARNAPPI   637
Fgram_10031                                       STLTRDLAPSAYFGRSAVVMASSSDDAAPVFFAMRALKISKIYTVGFRTPISLARNAPPI   637
MGG_12392                                         STLTRDLAPSAYFGCSAVVLASSSEDAASVIFALKALRVGKVFTVGFKTPPALATDL-EI   643
NCU_04298                                         STLTRDFAPSAYSGRAAVVLASNSDDAASSLLALKALMVSKIYTVGFKASPAVARDF-NL   638
Spoth2p4_46981                                    STLTRDFAPSAYFGCAAVVLASSAEDAASALFALKALKVGKVYTVGFKTPPAFGKDL-LI   637
Bcin_P100270                                      ATLTRDFAPSAYKDRAAIILSSNGEDAASVIFALRSLNIGKIYTIGFKAHGPLAV---GV   652
OIDMA_113116                                      ------FAPSAYKDRAAIILSNSGENAASVIFALRSLNIGKIYTVGFKALGPLAS---GV   461
TSTA_071040                                       ATLTRDYIPSAYRSRAVIILSSSQSDAAAAIFALRSLDVGTIYTVGFKASGPLAA---GL   568
Pc22g17290                                        ATLTREYVPSAYSSRAAIILASSQADASAAIFALRSLAVGTIYTVGFKASGPLAA---GL   604
Afu_4906460                                       ATLTRDYVPSAYRGRAAIILAGSESDASAAIYALRSLGVGSIYTVGFRATGPLAA---GL   616
AN10544                                           ATLTRDYVPSAYRGRAAIILAGSESEASASIFALRSLGVGAIYTVGFRATGPLAE---GL   577
An04g00790                                        ATLTRDYVPSAYRGRAAIILAGSESEASATIFALRSLGVGAIHTVGFRARGPLAE---GL   585
AOR_1_1674144                                     ATLTRDYVPSAYRGRAAIILAGSESEASAAIFALRSLGVGAIYTVGFRASGPLAD---GL   576
                                                          **** . :.::::    .*:  : *:  * :. :.*:**.    .
```

Figure S7. Alignment of protein sequences homologous to GaaX present in 18 Pezizomycetes species using Clustal omega (Sievers *et al.*, 2011). Missense mutations in *A. niger* GaaX and conservation in other species are highlighted. (Continued)

```
                                                                                             L607R
Zymosep_111379_mod_JGI_101685                     KRIGGLEELSR-------------------------IEEPVVVITALPARQAKCIAPVLT   579
Aurpu_var_pull_409643                             TPFRGLEDVKK-------------------------VEQPSVIISALRADKSLLVSPLLK   593
jgi|CocheC4_1|100143|replaces_COCCC4DRAFT_33348   SPVRSIEDVKR-------------------------LQQPFVIISALPAEKSLLVVPLLK   591
Tr_5296                                           EQFISMESLQRARSVAD-------------------NRAPFVIISALGRGKSNIVGMLLR   635
FoxG_05376                                        EPFISLESLQRARSVED-------------------DSAPFVIVSALGSEKSHLVGMLLR   678
Fgram_10031                                       EPFISLESLQRARSVEN-------------------DSAPFVIVSALGSDKSHLVGMLLR   678
MGG_12392                                         EPFTSLESLQRARSPSMNTAAGSTLNHKMDPGHQQQQLPPFVVVSALSAEKASIVGMVVR   703
NCU_04298                                         EPFDSLESMQKARTNDANPANQ--------------GASPFVVVSALAPEKSNLVGMLVR   684
Spoth2p4_46981                                    EPFTSLESIQRARTVGANGSNV-----------GTGTGSPFLVVSALGPEKSTLVGMLVR   686
Bcin_P100270                                      ESFTSMESVTK-------------------------SDIPFAIISALPPEKTHLVQPLLR   687
OIDMA_113116                                      EPFTSIESVQR-------------------------VDQPFAIISALPPEKSHLVQPLLR   496
TSTA_071040                                       EPFTSIQSVKL-------------------------VEQPFVIVSALPPEKSLLVQPLLR   603
Pc22g17290                                        EPFTSIQSVKR-------------------------VEQPFVIVSALPPEKSLLVQPLLR   639
Afu_4906460                                       EPFTSIQSVKL-------------------------VEQPFVMVSALPPEKSLLVQPLLR   651
AN10544                                           EPFTSIQSVKL-------------------------VEQPFVIVSALPPEKSLLVQPLLR   612
An04g00790                                        EPFTSIQSVKM-------------------------VEQPFVMVSALPPEKSLLVQPLLR   620
AOR_1_1674144                                     EPFTSIQSVKL-------------------------VEQPFVIVSALPPEKSLLVQPLLR   611
                                                    . .::.:                              *  :::**   ::  :  ::

                                                                               D604I
Zymosep_111379_mod_JGI_101685                     LLAEVGVKGTT----------RDDRARGVLLNLE---GKG-TSEAAEIAEHLGWKTFDAV   625
Aurpu_var_pull_409643                             HYGSHKQ--------------EQRRAAKVFLDLS--NGPR-KGDPVALAQTQGWAAYGIA   636
jgi|CocheC4_1|100143|replaces_COCCC4DRAFT_33348   HYRVAGRNGSPEGSPKALGAVGTKTAGKVFVDLA--SGPR-KVDTLEIATSAGWTAYGIA   648
Tr_5296                                           VFGEAGPPG-------------SSNSRKVFLNLEAAASQQ-KNDPVAVAEQSGFTAYDAA   681
FoxG_05376                                        AFGGVGPKG-------------TTNTKKVFLDLADGAVRK-SSDPGLIAERNGFAAYGAD   724
Fgram_10031                                       AFGGVGPKG-------------VTHTKKVFLDLADGAGRK-SSDPGLIAEKNGFAAYGAD   724
MGG_12392                                         LFGSQAGKPSVPTTANGMNGTPAAPQRRVFLDLSQPSGQRRGADPTLIAEQNGFAAYGLA   763
NCU_04298                                         VFGSRGSS----------------SSRKVFLDLADGSFPR-KGDPKVIAEQFGFAAYGAE   727
Spoth2p4_46981                                    LFGTRGSG---------------TDSRKVFLDLADRPASA-KGDPALIAERSGFAAYGAA   730
Bcin_P100270                                      YFN----GR------------DSRGQGKVFVDLA--HGPR-KGDPLAVAEGCGWTAYGVA   728
OIDMA_113116                                      HFSNGKEGQ------------NGAGLGKVFVDLS--NGPK-KGDPIKVAEQSGWTAYGVA   541
TSTA_071040                                       HYRSNGRAS------------PPSTR-----------GKR-KGDPLGVATSAGWTAYGID   639
Pc22g17290                                        HYGVNGRCS------------PPCTRGRVYLDLT--SGPR-KGDPLAVATRAGWTAYGVE   684
Afu_4906460                                       HYRSNGRTS------------PRSTRGKVYLDLTASSGER-KGDPVGVAVNAGWTAYGID   698
AN10544                                           HYRSTGRAS------------PPSTRGKVYLDLT--RGER-TGDPVSVAERSGWTAYGIE   657
An04g00790                                        HYRTSGRTS------------PPSTRGKVYLDLT--RGER-TGDPVGVAVRAGWTAYGIE   665
AOR_1_1674144                                     HYRTSGQTS------------PPSTRGKVYLDLT--RGER-TGDPVGVAVRAGWTAYGIE   656
                                                                                             :   :*   *: ::.

                                                          L676P   V648K
Zymosep_111379_mod_JGI_101685                     DVQCRFVAETLRQASFNHVASRL----QGDQACGEKRQYRGS*   663
Aurpu_var_pull_409643                             DVNAWTTVETLRLLVGQNVPYDFVRLAAGRSIY----------   669
jgi|CocheC4_1|100143|replaces_COCCC4DRAFT_33348   DVSAWTTVETIRKLVGQNVCYDFVRLACGRGHF----------   681
Tr_5296                                           DVAAFTTVESLRLLVGQNVPYSFVRLASGNLF-----------   713
FoxG_05376                                        DVAAFTTVESLRLLVGQNVPYSFVRLASGSHRYGV--------   759
Fgram_10031                                       DVAAFTTVESLRLLVGQNVPYSFVRLASGSHRYGA--------   759
MGG_12392                                         DTAAFATVETLRLLVGQNVPYSFVRLASGRPY-----------   795
NCU_04298                                         DVMAFTTVETLRLLVGQNVPYSFVRLASGRPMC----------   760
Spoth2p4_46981                                    DVTAFTTVETLRLLVGQNVPYSFVRLASGRTLF----------   763
Bcin_P100270                                      DTSAFTTVETLRLLVGQNVPYSFVRLASGRGLY----------   761
OIDMA_113116                                      DTSAFTTVETLRLLVGQNVPYSFVRLASGRGLY----------   574
TSTA_071040                                       DVNAWTTVETMRLLVGQNVPFDFVKMASGHGLY----------   672
Pc22g17290                                        DVSAWTTVETLRLLVGQNVPFDFVRMASGRPLF----------   717
Afu_4906460                                       EVTAWTTVETMRLLVGQNVPFDFVRMASGRIY-----------   730
AN10544                                           DVNAWTTVEMLRLLVGQNVPFDFVRMASYSLV-----------   689
An04g00790                                        DVDAWTTVETLRLLVGQNVPFDFVRMASGRVF-----------   697
AOR_1_1674144                                     DVNAWTTVETLRLLVGQNVPFDFVRMASGRVF-----------   688
                                                  :. .  ..* :*    ::*   :
```

Figure S7. Alignment of protein sequences homologous to GaaX present in 18 Pezizomycetes species using Clustal omega (Sievers *et al.*, 2011). Missense mutations in *A. niger* GaaX and conservation in other species are highlighted. (Continued)
